# Anticancer and cancer preventive activities of shogaol and curcumin from Zingiberaceae family plants in KG-1a leukemic stem cells

**DOI:** 10.1186/s12906-025-04829-7

**Published:** 2025-02-28

**Authors:** Pawaret Panyajai, Natsima Viriyaadhammaa, Sawitree Chiampanichayakul, Yasuhisa Sakamoto, Siriporn Okonogi, Toshiro Moroishi, Songyot Anuchapreeda

**Affiliations:** 1 Department of Medical Technology, Faculty of Associated Medical Sciences, Chiang Mai University, Chiang Mai, 50200 Thailand; 2 Cancer Research Unit of Associated Medical Sciences (AMS CRU), Faculty of Associated Medical Sciences, Chiang Mai University, Chiang Mai, 50200 Thailand; 3 Center of Excellence in Pharmaceutical Nanotechnology, Chiang Mai University, Chiang Mai, 50200 Thailand; 4 Center for Metabolic Regulation of Healthy Aging, Faculty of Life Sciences, Kumamoto University, Kumamoto, 860-8556 Japan; 5 Department of Pharmaceutical Sciences, Faculty of Pharmacy, Chiang Mai University, Chiang Mai, 50200 Thailand; 6 Department of Molecular and Medical Pharmacology, Faculty of Life Sciences, Kumamoto University, Kumamoto, 860-8556 Japan

**Keywords:** Zingiberaceae family, Cancer, Leukemia, Leukemia stem cell, Curcumin, Shogaol, WT1, Cancer prevention property, Apoptosis

## Abstract

**Background:**

Leukemic stem cells (LSCs) present a significant challenge in the treatment of leukemia in patients because they exhibit a drug-resistant phenotype, making them difficult to eliminate. Searching for a new anticancer drug is crucial for improving leukemia treatment. Plants from the Zingiberaceae family are frequently used in traditional medicines due to their safety and accessibility. This study explores the anticancer activity, cancer preventive properties, and apoptosis inducing mechanisms of active compounds derived from these plants.

**Methods:**

Ten crude ethanolic extracts from each plant of the Zingiberaceae family were obtained using maceration techniques. The cytotoxicity of all extracts anticancer was assessed in comparison to anticancer drugs (cyclophosphamide, cytarabine, doxorubicin, and idarubicin) using MTT assay on cancer cell lines (KG-1a, K562, A549, MCF-7, and HeLa) and peripheral blood mononuclear cells (PBMCs). Cancer prevention properties of the effective extracts and their active compounds were evaluated by measuring the levels of tumor necrosis factor-alpha (TNF-α), interleukin-2 (IL-2), and nitric oxide (NO) using commercial kits. Cell cycle and cell death analyses were conducted using flow cytometry. Moreover, the effects of effective extracts and their active compounds on WT1 and CD34 expressions, as well as the apoptosis mechanism induced by the active compounds in KG-1a cells, were determined by Western blotting.

**Results:**

The cytotoxicity tests revealed that crude ethanolic extracts from *Curcuma longa*, *C. zedoaria*, and *Zingiber officinale* exhibited effective cytotoxicity against cancer cell lines while demonstrating lower impact on PBMCs. The active compounds of *C. longa* and *C. zedoaria* are curcuminoids, while those in *Z. officinale* are shogaol and gingerol. Notably, the IC_20_ values of curcuminoids and shogaol exhibited cancer prevention properties and reduced WT1 protein expression, thereby inhibiting cell proliferation. Furthermore, shogaol and curcumin demonstrated the ability to arrest the cell cycle at the G_2_/M phase and induce apoptosis through the Akt pathway.

**Conclusion:**

These findings highlight shogaol and curcumin as promising compounds for leukemia treatment.

**Supplementary Information:**

The online version contains supplementary material available at 10.1186/s12906-025-04829-7.

## Introduction

Chemotherapy is one of the effective methods for leukemia treatments. However, most of the chemotherapies function by obstructing DNA replication, a mechanism that affects both cancer and normal cells alike, leading to unpleasant side effects for patients. Plants from the Zingiberaceae family are commonly used in traditional medicines and as cooking ingredients in Southeast Asia. Most plants in this family are extensively employed in traditional remedies to address issues such as stomachaches, hemorrhoids, blood circulation, and muscular pain relief. They are also used in herbal compress balls for massages. Moreover, rhizomes and leaves of these plants are used as spices and cooking ingredients. Previous reports have highlighted the diverse biological activities from this family [1–4], presenting an intriguing avenue for developing anticancer drugs with reduced side effects, improved accessibility, and enhanced safety.

Unregulated inflammation is often associated with the development and progression of cancer since inflammatory response can damage the DNA of normal cells. This damage increases the risk of mutations that eventually contribute to cancer [5–7]. Several active compounds from plants, such as sulforaphane, lycopene, curcumin, shogaol, and resveratrol, exhibited cancer prevention properties by suppressing the production of inflammatory cytokines and nitric oxide (NO), while enhancing the phagocytic activity of macrophages [8–12]. Thus, suppression of unregulated inflammation represents a potential strategy for preventing cancer initiation [13].

Currently, numerous studies have highlighted leukemic stem cells (LSCs) as a significant challenge in patients with leukemia. These cells exhibit surface marker phenotypes CD34^+^/CD38^−^ and share characteristics with hematopoietic stem cells (HSCs) [14, 15]. Additionally, they demonstrated greater resistance against chemotherapeutic drugs compared to normal leukemic cells, making their elimination crucial for improving treatment outcomes. Wilms’ tumor 1 (WT1) plays an active role in cell growth and development during normal hematopoiesis [16–20]. However, the overexpression of WT1 protein can induce leukemic cell proliferation. Consequently, the overexpression of the *WT1* gene has been used as a biological marker for diagnosing and evaluating minimal residual disease (MRD) in leukemia [21]. Furthermore, the elevated expression of WT1 protein is associated with poor prognosis and worse long-term outcomes in acute myeloid leukemia (AML) [21–23], often leading to relapse [24]. These findings suggested that WT1 plays a crucial role in leukemogenesis, as it is present in both leukemia cells and leukemic stem cells. Simultaneously, many studies have revealed the crucial role of the Hippo signaling pathway in regulating organ size, tissue homeostasis, and cell proliferation. Dysregulation of this pathway also contributes to various diseases, including cancer [25, 26]. In leukemia, Yes-associated protein (YAP) plays important roles in proliferation and leukemogenesis, particularly in chronic myeloid leukemia (CML) [27]. YAP is a key molecule in Hippo signaling pathway. Furthermore, inhibiting YAP function has been shown to limit cell proliferation and induce apoptosis in HL-60 cells [28]. Thus, targeting the Hippo signaling pathway may offer novel opportunities for overcoming resistance in leukemic stem cells and advancing leukemia treatment.

While numerous reports have explored the biological activities of compounds of plants from the Zingiberaceae family, there is less information regarding the effects of extracts or active compounds from this family on WT1 protein expression and the Hippo signaling pathway in LSCs. To investigate this unexplored aspect, this study aims to evaluate the cytotoxicity of ten plant extracts from the Zingiberaceae family in a leukemic stem cell line, comparing their effects to those on other cancer cell lines and peripheral blood mononuclear cells (PBMCs). Additionally, we aim to explore the cancer preventive properties of extracts from candidate plants and investigate the apoptotic mechanisms induced by active compounds in KG-1a leukemic stem cell line. By assessing their impact on pathways associated with leukemogenesis, this study aims to provide new insights into the therapeutic potential of these plants for leukemia treatment.

## Materials and methods

### Crude ethanolic extraction

In June 2019, fresh rhizomes of *Alpinia galanga*, *Boesenbergia rotunda*, *Curcuma aeruginosa*, *C. longa*, *C. mangga*, *C. zedoaria*, *Kaempferia parviflora*, *Zingiber montanum*, *Z. officinale*, and *Z. ottensii* were collected from a local garden in Chiang Mai, Thailand.

The authors confirm that all methods involving the plants, and their materials complied with relevant institutional, national, and international guidelines and legislation. Additionally, these plants are common medicinal plants in Thailand and are widely used in Thai traditional medicine. They are not at risk of extinction. All plant materials were identified and authenticated by Wannaree Charoensup, Chiang Mai University, Chiang Mai, Thailand. Their voucher specimens (deposition no. 009245 for *A. galanga*, 009724 for *B. rotunda*, 0023261 for *C. aeruginosa*, 023356 for *C. longa*, 0023362 for *C. mangga*, 0023369 for *C. zedoaria*, 0023370 for *K. parviflora*, 004581 for *Z. montanum*, 0023361 for *Z. officinale*, 000109 for *Z. ottensii*) were deposited in the Herbarium, the Faculty of Pharmacy, Chiang Mai University, Thailand.

Plant collection was done under the license of the Ghana Forestry Commission, according to the guidelines of the IUCN Policy Statement on Research Involving Species at Risk of Extinction and the Convention on the Trade in Endangered Species of Wild Fauna and Flora.

The ethanolic extracts of the fresh rhizomes were obtained using a modified maceration technique based on a previous study [29]. Briefly, each rhizome was dried and ground into a powder. Then, the powder was macerated with 95% ethanol at a ratio of 1:3 for 48 h. The filtrates were collected by filtration technique using a filter cloth, and the residue was subjected to maceration with 95% ethanol at a ratio of 1:3 for an additional 48 h, repeated three times. The filtrates were evaporated to dryness using a rotary evaporator, and the resulting extracts were stored in light-resistant containers within a −20 °C freezer.

### Active compounds

Standard curcuminoids (80% pure curcumin, 17% demethoxycurcumin, and 3% bisdemethoxycurcumin), shogaol, and gingerol were purchased from Sigma-Aldrich (MA, USA). The “in-house” curcuminoids refer to compounds purified in our laboratory. These compounds were extracted from natural sources such as the rhizomes of *C. longa* and *C. zedoaria*, subsequently purified in our laboratory using column chromatography. The purified compounds were then analyzed using HPLC to confirm their identity and purity, as described previously [30, 31]. The in-house curcumin, demethoxycurcumin, and bisdemethoxycurcumin, after column chromatography purification, exhibited 100% purity in each compound, as demonstrated in a previous study [32].

### Cell culture

Five cancer cell lines (KG-1a, K562, A549, MCF-7, and HeLa) were used as human cancer cell line models in this study. KG-1a cells were cultured in IMDM (Iscove’s Modified Dulbecco’s Medium) medium (Invitrogen™, MA, USA) supplemented with 20% fetal bovine serum (Capricorn Scientific, Ebsdorfergrund, Germany), 100 units/mL penicillin and 100 µg/mL streptomycin (Invitrogen™, MA, USA). K562 cells were cultured in RPMI (Roswell Park Memorial Institute)-1640 medium (Invitrogen™, MA, USA) supplemented with 10% fetal bovine serum, 2 mM L-glutamine (Invitrogen™, MA, USA), 100 units/mL penicillin and 100 µg/mL streptomycin. A549, MCF-7, HeLa, and RAW264.7 cells were cultured in DMEM (Dulbecco’s Modified Eagle Medium) medium (Invitrogen™, MA, USA) supplemented with 10% fetal bovine serum 100 units/mL penicillin and 100 µg/mL streptomycin (Invitrogen™, MA, USA). All cancer cell lines were cultured at 37 °C in a humidified incubator with 5% CO_2_.

### Isolation of PBMCs

PBMCs were isolated from blood samples using the Ficoll-Hypaque technique. Initially, 10–20 mL of blood was collected in a heparin tube at a ratio of 1:500 from a minimum of three healthy volunteers. Subsequently, the blood samples were diluted with Phosphate buffer saline (PBS), pH 7.4, at a ratio of 1:1 to reduce viscosity. Histopaque-1077 (Sigma-Aldrich, MA, USA) was then underlaid the diluted sample and centrifuged at 400×g for 30 min. The resulting PBMCs were collected, washed with PBS, pH 7.4, and prepared for further experiments.

### MTT test

MTT (3-(4,5-dimethylthiazol-2-yl)-2,5-diphenyltetrazolium bromide) assay was used to investigate the cytotoxicity of the test samples. KG-1a (1.5 × 10^4^ cells/well), K562 (1.0 × 10^4^ cells/well), PBMCs (1.0 × 10^5^ cells/well), A549 (5.0 × 10^3^ cells/well), MCF-7 (5.0 × 10^3^ cells/well), and HeLa (5.0 × 10^3^ cells/well) were seeded into a 96-well plate and incubated overnight at 37 °C with 5% CO_2_. Subsequently, the cells were treated with various concentrations of crude ethanolic extract (0–100 µg/mL), active compounds (0–100 µg/mL), or chemotherapeutic drugs as positive controls: cyclophosphamide (0–400 µg/mL), cytarabine (0–100 µg/mL), doxorubicin (0–1,000 ng/mL), and idarubicin (0–1,000 ng/mL), with 0.4% DMSO as the vehicle control (VC), for 48 h. After that, 5 mg/mL of MTT dye solution (Sigma-Aldrich, MA, USA) was added and incubated for 4 h. The produced formazan crystals were dissolved in 200 µL of DMSO (Sigma-Aldrich, MA, USA); then, the optical density (OD) was measured using an ELISA plate reader (Metertech, Taipei, Taiwan) at 578 nm with a reference wavelength of 630 nm. The percentage of surviving cells was calculated from the absorbance values of the test and control wells using the following Equation. 

1 $$\:\text{\%}\:\text{C}\text{e}\text{l}\text{l}\:\text{v}\text{i}\text{a}\text{b}\text{i}\text{l}\text{i}\text{t}\text{y}\:=\:\frac{{\text{OD}}_{\text{s}\text{a}\text{m}\text{p}\text{l}\text{e}}}{{\text{OD}}_{\text{vehicle control}}}\:\times\:\:100$$

where OD_sample_ is a mean absorbance in test well and OD_vehicle control_ is a mean absorbance in VC well. The average percentage of surviving cells at each concentration obtained from triplicate experiments was plotted as a dose-response curve. Inhibitory concentration values at 50% growth (IC_50_ value) were defined as the lowest concentration of the test sample that inhibited cell growth by 50% compared to the untreated control.

To calculate IC_50_ values, a dose-response curve was fitted using non-linear regression analysis, as described in the GraphPad Prism guidelines (https://www.graphpad.com/support/faq/how-to-determine-an-icsub50sub/). The IC_50_ value was obtained by determining the point at which the curve intersected 50% growth inhibition. Non-linear regression graphs of the dose-response curve for each compound are provided in the supplementary data file (Figs. S1–S8).

The selectivity index (SI) of active compounds and chemotherapeutic drugs was calculated for using the following Eq. 

2 $$\:\text{S}\text{e}\text{l}\text{e}\text{c}\text{t}\text{i}\text{v}\text{i}\text{t}\text{y}\:\text{i}\text{n}\text{d}\text{e}\text{x}\:\left(\text{S}\text{I}\right)\:=\:\frac{{\text{IC}}_{\text{50 N}}}{{\text{IC}}_{\text{50 C}}}$$

where IC_50 N_ is IC_50_ for normal cells and IC_50 C_ is IC_50_ for cancer cells which were treated with the same compounds in both cells.

### Trypan blue exclusion test

This test was used to confirm the cytotoxicity of the test samples. The cancer cells after exposure to crude ethanolic extracts and active compounds for 48 h were harvested and washed with ice-cold PBS, pH 7.4 for 3 times. Then, the cells were resuspended with PBS, pH 7.4. The cell suspensions were diluted with PBS, pH 7.4 at the appropriate dilution before being mixed with 0.2% trypan blue solution at 1:2 dilution for cell count on a hemocytometer. From this test, the viable cells and the dead cells could also be obviously detected.

### Cancer prevention properties

For the measurement of tumor necrosis factor-alpha (TNF-α) and NO, RAW264.7 cells were pre-treated with plant extracts and active compounds at a non-cytotoxic dose (IC_20_) (Table S9) or 1 µg/mL of dexamethasone (positive control) in a 24-well plate for 2 h. Subsequently, 1 µg/mL of lipopolysaccharide (LPS) (Cat No. 00497693, Invitrogen™, MA, USA) was added to each well, and the cells were incubated for an additional 24 h to allow sufficient time for the compounds to exert measurable anti-inflammatory effects while maintaining cell viability. Afterward, supernatants were collected, and the levels of TNF-α and NO were measured using the Mouse TNF alpha Uncoated ELISA Kit (Invitrogen™, ThermoFisher Scientific, MA, USA) and the Total Nitric Oxide Assay Kit (Invitrogen™, ThermoFisher Scientific, MA, USA), respectively, according to the manufacturer’s instructions.

For the measurement of interleukin-2 (IL-2), human PBMCs were pre-treated with plant extracts and active compounds at the IC_20_ concentration (Table S9) or 1 µg/mL of dexamethasone (positive control) in a 24-well plate for 2 h. Subsequently, 20 µL/mL of phytohemagglutinin (PHA) (Cat. No. 10576015, Thermo Fisher Scientific, MA, USA) was added to each well, and the cells were incubated for an additional 24 h. After that, supernatants were collected, and the levels of IL-2 were measured by the Human IL-2 Uncoated ELISA Kit (Invitrogen™, ThermoFisher Scientific, MA, USA), according to the manufacturer’s instructions.

The percentage of NO or cytokine inhibitions were calculated using the following equation:

3 $${\rm{\% }}\,{\rm{NO}}\,{\rm{or}}\,{\rm{cytokine}}\,{\rm{inhibition}}\, = \,{{{\rm{A}} - {\rm{B}}} \over {\rm{A}}}\, \times \,100$$

where A is the level of cytokine or NO without treatment, and B is the level of cytokine or NO with treatment.

### Cell cycle and apoptosis assay

After treating KG-1a cells with active compounds at various concentrations for 48 h, the cells were pulsed with 10 µM BrdU (Sigma-Aldrich, MA, USA) for 20 min at 37 °C in an incubator with 5% CO_2_. Subsequently, the cells were harvested, washed with ice-cold PBS, pH 7.4 three times, and then fixed with 70% ethanol in PBS, pH 7.4 at −30 °C. Following fixation, the cells were washed and resuspended in 2 N HCl/0.5% Triton X-100 for 30 min at room temperature (RT). Then, 0.1 M sodium tetraborate decahydrate was added for 2 min at RT to neutralize the acid. Subsequently, the cells were stained with FITC-anti-BrdU antibody for 30 min at RT. After washing, the cells were resuspended in propidium iodide (PI) (Dojindo, Kumamoto, Japan) in PBS, pH 7.4 at a ratio of 1:1,000. The cell cycle phases were detected using a flow cytometer (CytoFLEX S, Beckman Coulter, CA, USA) and analyzed with the CytExpert program (Beckman Coulter, CA, USA).

The flow cytometry data were analyzed to assess both apoptosis and cell cycle distribution. Apoptosis was evaluated by determining the percentage of cells in the sub-G_1_ phase using the Nicoletti assay. For cell cycle analysis, populations in the G_1_, S, and G_2_/M phases were calculated after excluding the sub-G_1_ population to avoid interference from apoptotic cells, allowing for a clearer assessment of cell cycle arrest in different phases. Data were presented in separate graphs for the sub-G_1_ population and cell cycle phase distributions.

### Cell death analysis

This test was used to confirm the cytotoxicity of crude ethanolic extracts and active compounds. KG-1a cells after exposure to crude ethanolic extracts and active compounds for 48 h were harvested and washed with ice-cold PBS, pH 7.4 for 3 times. Then, the cells were resuspended with PBS, pH 7.4. The cell suspensions were diluted with PBS, pH 7.4 at the appropriate dilution before being mixed with 0.2% trypan blue solution at 1:2 dilution for cell count on a hemocytometer. From this test, the viable cells and the dead cells could also be obviously detected.

Additionally, PI staining was used to investigate the cytotoxicity of the active compounds. KG-1a cells after exposure to the active compounds with various concentration were harvested at different incubation times and washed with ice-cold PBS, pH 7.4 for 2 times. Then, the cells were resuspended with PI in PBS, pH 7.4 at a ratio of 1:10,000. The percentage of dead cells was detected by flow cytometry (CytoFLEX S, Beckman Coulter, CA, USA) and analyzed by CytExpert program (Beckman Coulter, CA, USA).

Both tests provided similar results regarding cell viability and cell death analysis.

### Western blotting

KG-1a cells (1.5 × 10^5^ cells/mL) were treated with compounds or extracts at different incubation times. Then, the cells were harvested and the whole proteins were extracted using a RIPA buffer (25 mM Tris-base, pH 7.6, 0.1% SDS, 1% Triton X-100, 150 mM NaCl, and 1 mM EDTA). The protein concentration was measured using the Folin-Lowry or Bradford assay. The whole protein lysates (30 µg/lane) were then separated through 7.5% SDS-PAGE under reducing conditions and then transferred to PVDF membranes. The membranes were blocked in 5% skim milk in PBS, pH 7.4 at RT for 2 h, then, target proteins were probed with specific primary antibodies (anti-WT1, anti-CD34, anti-GAPDH, anti-cleaved caspase-3, anti-cleaved PARP, anti-phospho-Akt, anti-phospho-c-Jun, anti-phospho-SAPK/JNK, and anti-YAP/TAZ) indicated in Table S10 which diluted at a ratio of 1:1,000 in PBS, pH 7.4 at RT for 2 h. After incubation, the membranes were rinsed 6 times with 0.1% Tween-20 (Sigma-Aldrich, MA, USA) in PBS, pH 7.4 (PBS-T), each time for 5 min. The reaction was followed by HRP-conjugated secondary antibody at 1:20,000 dilution in PBS, pH 7.4 at RT for 2 h, then, the membranes were rinsed 6 times for 5 min each with PBS-T. The proteins were shown using Immobilon Forte Western HRP substrate (Millipore, MA, USA). Finally, the protein band signal was quantified by using a scan densitometer (Bio-Rad Laboratories, CA, USA).

### Statistical analysis

All experiments were performed in triplicate except the apoptosis mechanism in KG-1a cells. The average of triplicate experiments and standard derivation (SD) were used for quantification. The levels of cell populations were compared to VC in each experiment. The results are shown as mean ± S.D. The SPSS statistics software ver. 22 (SPSS Inc., IL, USA) was used for statistical analysis. Differences between the means of each sample were analyzed by one-way analysis of variance (one-way ANOVA), followed by LSD post-hoc analysis. Statistical significance was considered at *p* < 0.05.

## Results

### Cytotoxicity of crude ethanolic extracts on cancer cell lines and PBMCs

The results indicated that each crude ethanolic extract exhibited various levels of cytotoxicity across different cancer cell lines and PBMCs. Notably, the crude ethanolic extracts of *C. longa*, *C. zedoaria*, and *Z. officinale* demonstrated good cytotoxicity in KG-1a cells, with the IC_50_ values of 24.59 ± 1.40, 24.72 ± 3.39, and 21.70 ± 1.83 µg/mL, respectively. According to National Cancer Institute (NCI) criteria, plants with an IC_50_ of ≤ 30 µg/mL are considered promising candidates for further investigation in cancer treatment [33, 34]. Moreover, these three extracts also exhibited substantial cytotoxicity in other cancer cell lines, except A549, while demonstrating low cytotoxicity in PBMCs (Table 1). Raw data for the IC_50_ values, obtained from non-linear regression graphs performed in triplicate, are provided in the supplementary file (Figs S1–S6 and Tables S1–S6).

**Table 1:** IC_50_ values of crude ethanolic extracts from Zingiberaceae plants on cancer cell lines and PBMCs after incubation for 48 h

Crude ethanolic extracts	IC_50_ (µg/mL)					
	KG-1a	PBMCs	K562	A549	HeLa	MCF-7
*A. galanga*	> 100	42.11 ± 3.84	49.10 ± 6.13	90.24 ± 3.75	52.51 ± 6.66	46.34 ± 3.02
*B. rotunda*	38.46 ± 0.20	39.83 ± 2.27	27.83 ± 4.49	51.60 ± 5.54	30.08 ± 5.52	38.68 ± 5.79
*C. aeruginosa*	74.80 ± 2.62	79.11 ± 3.92	64.70 ± 3.63	> 100	57.57 ± 8.16	66.05 ± 2.95
*C. longa*	24.59 ± 1.40	76.30 ± 9.13	26.32 ± 2.76	80.10 ± 9.23	20.34 ± 0.39	19.56 ± 3.08
*C. mangga*	83.97 ± 2.04	> 100	48.76 ± 1.58	> 100	66.55 ± 1.45	64.32 ± 5.76
*C. zedoaria*	24.72 ± 3.39	42.26 ± 3.15	41.17 ± 3.33	> 100	24.68 ± 1.91	18.83 ± 2.41
*K. parviflora*	29.34 ± 5.09	46.77 ± 1.48	52.38 ± 7.18	71.33 ± 2.57	15.88 ± 0.97	24.48 ± 1.17
*Z. montanum*	46.65 ± 1.82	56.25 ± 3.41	66.14 ± 3.79	> 100	58.92 ± 5.15	36.05 ± 6.36
*Z. officinale*	21.70 ± 1.83	69.38 ± 10.50	37.76 ± 5.40	86.37 ± 6.81	12.38 ± 1.49	11.78 ± 1.73
*Z. ottensii*	50.66 ± 8.14	> 100	> 100	> 100	> 100	26.69 ± 8.35

Table 1 IC_50_ values of crude ethanolic extracts from Zingiberaceae plants on cancer cell lines and PBMCs after incubation for 48 h.

### Cytotoxicity of active compounds in KG-1a cells and PBMCs

The active compounds of *C. longa* and *C. zedoaria* are curcuminoids, including curcumin, demethoxycurcumin, and bisdemethoxycurcumin, while *Z. officinale* contains shogaol and gingerol. The purity of all curcuminoids was verified using HPLC, as reported in previous studies [30, 31]. HPLC analysis confirmed that in-house curcumin, demethoxycurcumin, and bisdemethoxycurcumin achieved 100% purity, as showed in a previous study [32]. Cytotoxicity analysis using MTT assay indicated that shogaol and three curcuminoids (curcumin, demethoxycurcumin, and bisdemethoxycurcumin) demonstrated significant cytotoxicity against KG-1a cells, with IC_50_ values of 2.99 ± 0.01, 10.52 ± 0.12, 11.28 ± 0.85, and 17.91 ± 0.99 µg/mL, respectively. Furthermore, all active compounds exhibited higher efficacy in cytotoxicity compared to cytarabine and cyclophosphamide against KG-1a cells. When evaluating cytotoxicity in PBMCs, all active compounds and chemotherapeutic drugs (cyclophosphamide, cytarabine, doxorubicin, and idarubicin) demonstrated lower cytotoxicity in PBMCs than in KG-1a cells, except for bisdemethoxycurcumin (Table 2). Raw data for the IC_50_ values, obtained from non-linear regression graphs performed in triplicate, are provided in the supplementary data (Figs S7–S8 and Tables S7–S8).

SI represents the ratio of the toxic concentration to the effective bioactive concentration of a sample. Ideally, compounds or drugs with a high SI value have a relatively high toxic concentration and a very low active concentration [35, 36]. In this study, the SI calculation revealed that shogaol, gingerol, curcumin, demethoxycurcumin, doxorubicin, and idarubicin had index values greater than 1, indicating that these compounds and drugs exhibited greater specificity towards KG-1a cells compared to normal PBMCs (Table 2).

**Table 2:** IC_50_ values and SI of active compounds and chemotherapeutic drugs in KG-1a cells and PBMCs after incubation for 48 h

Compounds and drugs	IC_50_		Selectivity index (SI)
	KG-1a	PBMCs	
Shogaol (µg/mL)	2.99 ± 0.01	9.18 ± 0.82	3.07
Gingerol (µg/mL)	76.77 ± 1.28	> 100	> 1.30
Curcumin (µg/mL)	10.52 ± 0.12	13.78 ± 0.82	1.31
Demethoxycurcumin (µg/mL)	11.28 ± 0.85	13.87 ± 0.28	1.23
Bisdemethoxycurcumin (µg/mL)	17.91 ± 0.99	11.64 ± 1.25	0.65
Cyclophosphamide (µg/mL)	> 400	> 400	> 1
Cytarabine (µg/mL)	> 100	> 100	> 1
Doxorubicin (ng/mL)	652.18 ± 142.06	> 1,000	> 1.53
Idarubicin (ng/mL)	31.27 ± 5.04	> 1,000	> 31.98

### Cancer prevention properties and cytotoxic effects of crude ethanolic extracts and their active compounds in cancer cell line and PBMCs

To investigate cancer prevention properties of plant extracts compared to their active compounds, NO and pro-inflammatory cytokines (IL-2 and TNF-α) were investigated in RAW264.7 cells and normal PBMCs after inflammatory induction and treated with IC_20_ value of each extract and active compound (Table S9). A non-cytotoxic dose is used to avoid cell death when determining target proteins in live cells. The result showed that all compounds did not show cytotoxicity on RAW264.7 and PBMCs after incubation for 24 h (Figs. S10 and S11). Each compound and extract had a different inhibitory effect on NO and cytokines.

**Fig. 1:**
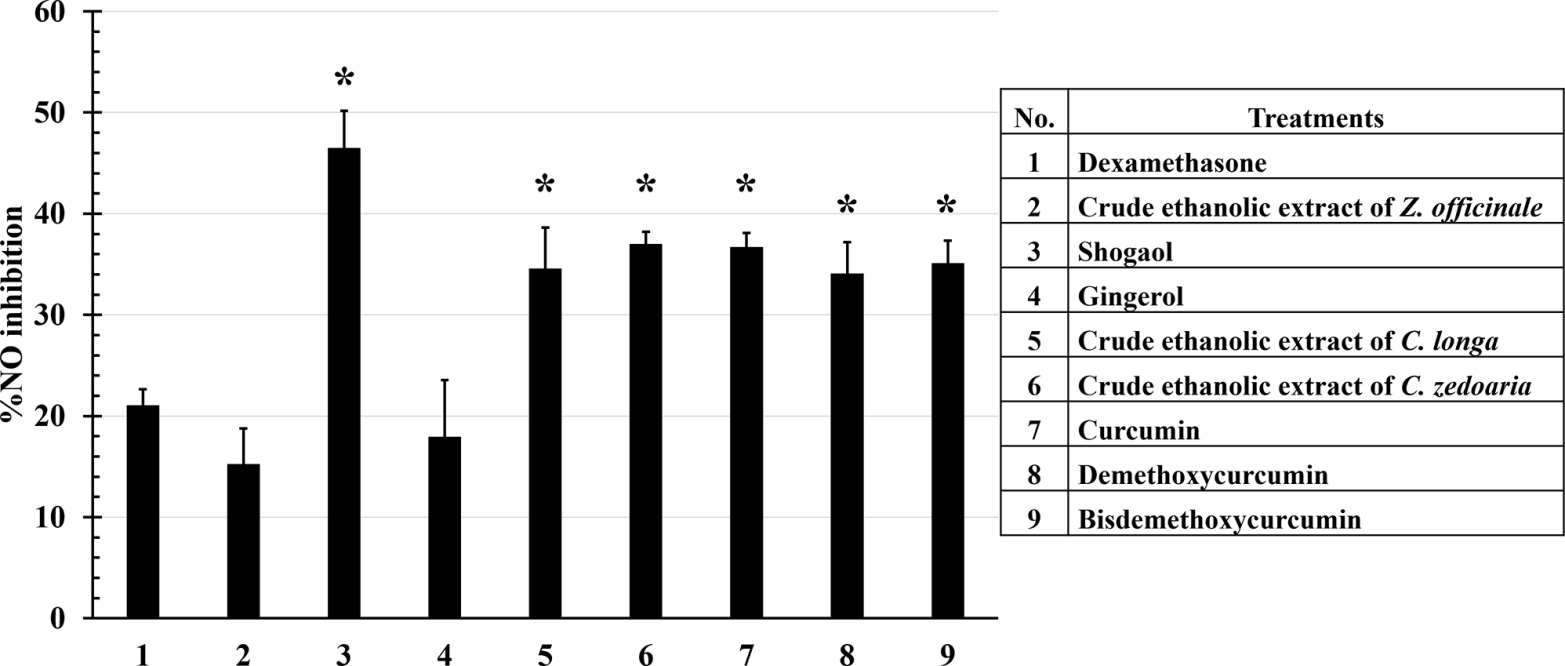
Inhibitory effects of crude ethanolic extracts and their active compounds on NO production. LPS-activated RAW264.7 cells were incubated with crude ethanolic extracts and their active compounds for 24 h. The supernatants were collected and measured the levels of NO were measured by NO assay. Shogaol, crude ethanolic extracts of *C. longa* and *C. zedoaria*, and all curcuminoids significantly reduced NO production compared to dexamethasone (positive control), while the crude extract of *Z. officinale* and gingerol had no effect. Data are the mean ± SD (*n* = 3); *p < 0.05 vs. positive control

Compared to dexamethasone (positive control), all compounds and extracts inhibited NO release by more than 20% compared to positive control, except crude ethanolic extract of *Z. officinale* and gingerol. When comparing the activity of the crude ethanolic extract to its active compounds, gingerol showed no significant difference from the crude ethanolic extract of *Z. officinale*. In contrast, shogaol exhibited significantly higher activity, inhibiting NO by 46.46 ± 3.71%, which was notably greater than that of gingerol and its crude ethanolic extract, and even more effective than dexamethasone. Regarding *C. longa*, and *C. zedoaria*, all curcuminoids and their crude ethanolic extract showed no significant difference in NO inhibitory activity, inhibiting approximately 35% (Fig. 1).

**Fig. 2:**
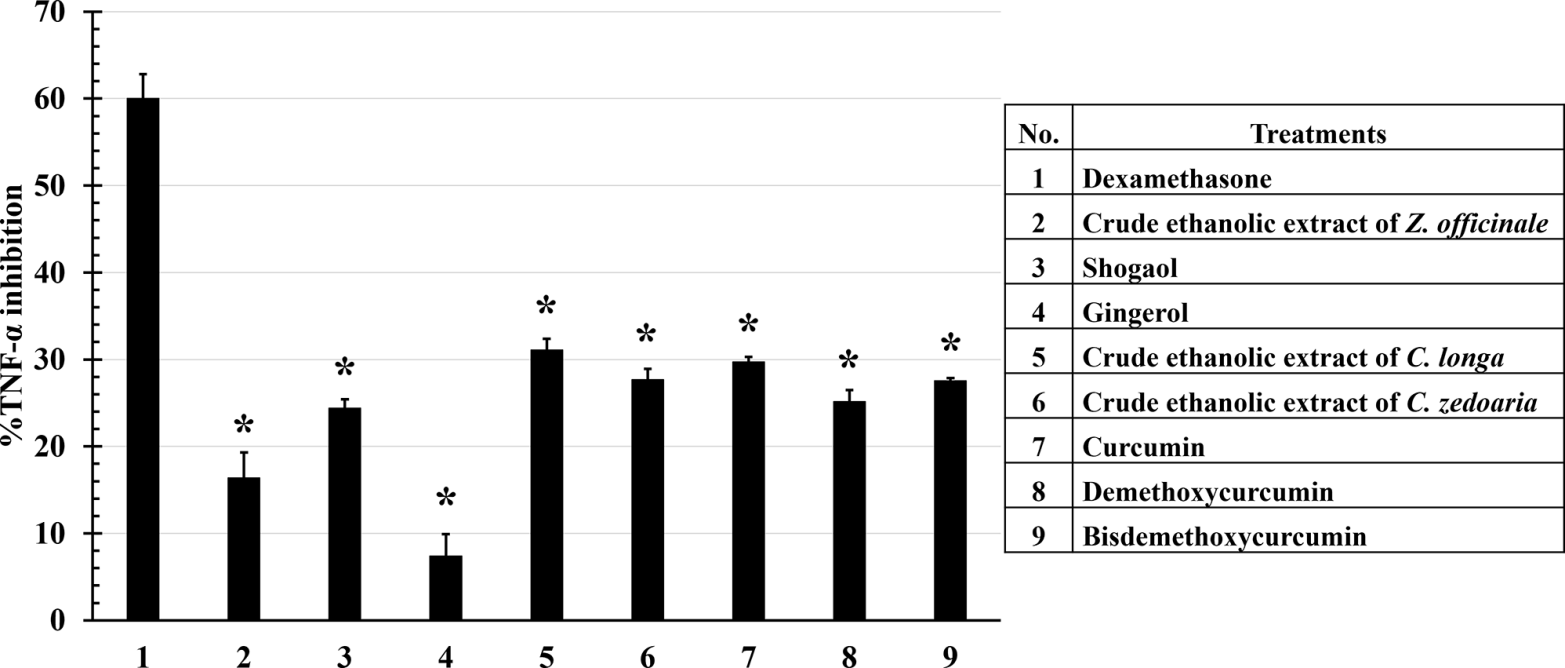
Inhibitory effects of crude ethanolic extracts and their active compound on TNF-α production. LPS-activated RAW264.7 cells were incubated with crude ethanolic extracts and their active compounds for 24 h. The supernatants were collected and measured the levels of TNF-α by ELISA kit. Most compounds and crude ethanolic extracts inhibited TNF-α release by more than 20%, with the crude ethanolic extract of *Z. officinale* and gingerol showing less than 20% inhibition. All treatments showed significantly lower activity than dexamethasone (positive control). Data are the mean ± SD (*n* = 3); *p < 0.05 vs. positive control

While most compounds and extracts inhibited TNF-α release by more than 20%, all showed significantly lower activity than the positive control. However, only gingerol showed less than 10% inhibitory activity. When comparing the activity of the crude ethanolic extract to their active compounds, shogaol exhibited higher inhibitory activity (24.41 ± 0.99%) compared to the crude ethanolic extract of *Z. officinale* and gingerol. Regarding *C. longa* and *C. zedoaria*, all curcuminoids and their crude ethanolic extracts showed no significant difference in TNF-α inhibitory activity with inhibition of approximately 25% or more (Fig. 2).

**Fig. 3:**
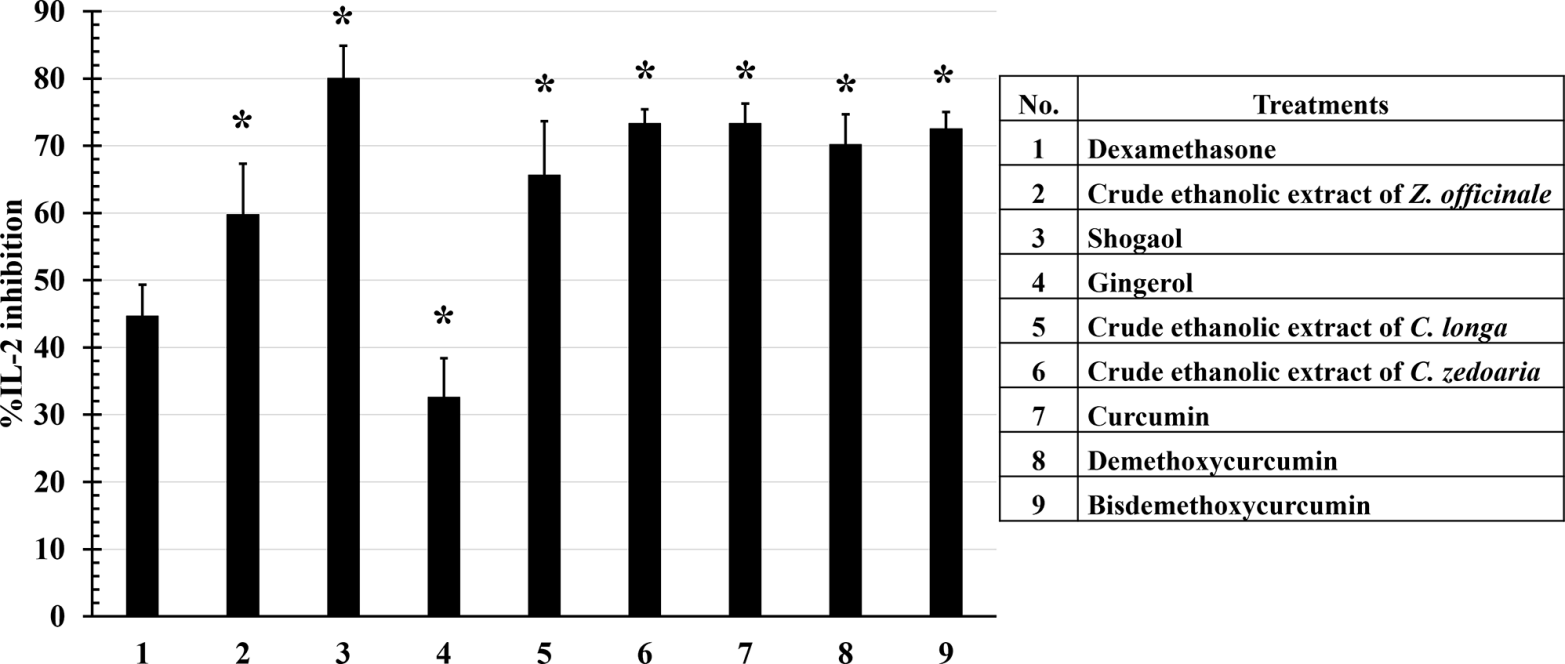
Inhibitory effects of crude ethanolic extracts and their active compound on IL-2 production. PHA-activated PBMCs were incubated with crude ethanolic extracts and their active compounds for 24 h. The supernatants were collected and measured the levels of IL-2 by ELISA kit. All compounds and extracts inhibited IL-2 release by more than 20% compared to dexamethasone (positive control). Data are the mean ± SD (*n* = 3); *p < 0.05 vs. positive control

All compounds and extracts also demonstrated effective inhibitory activity for IL-2 release when compared to positive control, with more than 20% inhibitory activity. However, while most crude ethanolic extracts and active compounds exhibited significantly higher inhibitory activity than positive control, gingerol showed significantly lower inhibitory activity. Comparing the activity between crude ethanolic extract and their active compounds, shogaol exhibited a significantly higher inhibitory activity by 80.04 ± 4.82% when compared to the crude ethanolic extract of *Z. officinale* and gingerol. For *C. longa* and *C. zedoaria*, all curcuminoids and their crude ethanolic extracts showed no significant difference in TNF-α inhibitory activity, which was suppressed by approximately more than 60% (Fig. 3).

### Effect of crude ethanolic extracts and active compounds on WT1 and CD34 protein expression in KG-1a cells

**Fig. 4:**
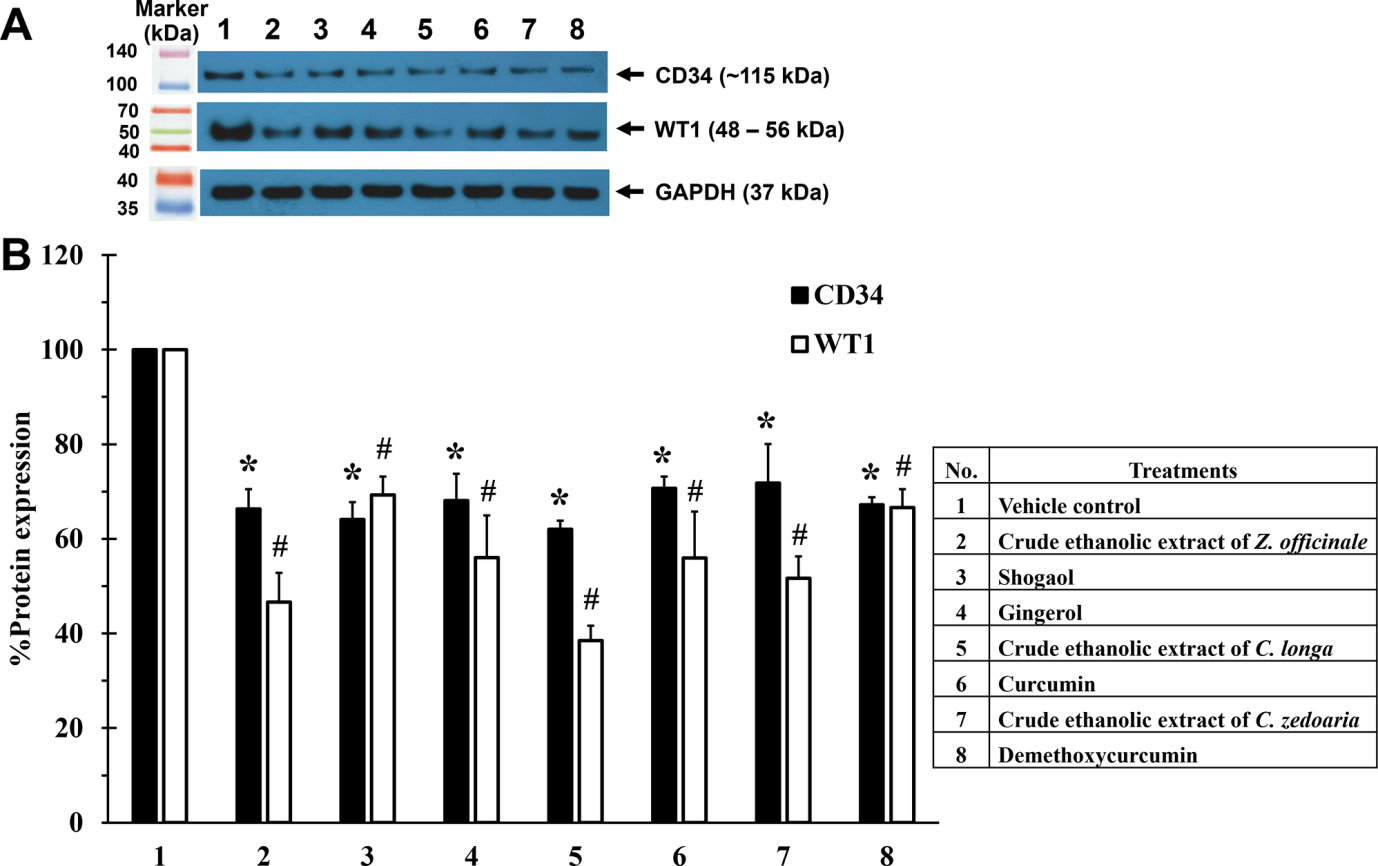
Effect of crude ethanolic extracts and their active compounds treatments on CD34 and WT1 protein expressions in KG-1a cells. KG-1a cells were treated with crude ethanolic extracts and their active compounds with IC_20_ concentrations for 48 h. CD34 and WT1 protein levels were determined by Western blotting. (**A**) Representative images and (**B**) representative bar graph of inhibitory effects of crude ethanolic extracts and their active compounds on WT1 and CD34 protein expressions in KG-1a cells for 48 h. The levels of protein were normalized using GAPDH protein levels. Full-length blots are presented in Supplementary file (Figs. S14–S16). All compounds and extracts inhibited WT1 and CD34 expressions significantly by more than 20% compared to VC. Data are the mean ± SD (*n* = 3); *p < 0.05 vs. VC

Although bisdemethoxycurcumin demonstrated potential cancer-prevention properties, it was excluded from subsequent experiments due to low SI, suggesting it had limited specificity for KG-1a cells and diminished therapeutic potential. After treatments with IC_20_ concentrations of the crude ethanolic extract of *Z. officinale*, shogaol, gingerol, the crude ethanolic extract of *C. longa*, curcumin, the crude ethanolic extract of *C. zedoaria*, and demethoxycurcumin, (Table S9) the result showed that WT1 protein levels were significantly decreased by 53.40 ± 6.21, 30.71 ± 3.90, 43.98 ± 8.93, 61.50 ± 3.11, 44.05 ± 9.81, 48.33 ± 4.62, and 33.37 ± 3.87%, respectively when compared to VC (Figs. 4A and B).

Additionally, the CD34 protein levels after treatment with the crude ethanolic extract of *Z. officinale*, shogaol, gingerol, the crude ethanolic extract of *C. longa*, curcumin, the crude ethanolic extract of *C. zedoaria*, and demethoxycurcumin at IC_20_ concentrations (Table S9) were significantly decreased by 33.70 ± 4.21, 35.93 ± 3.70, 31.95 ± 5.70, 37.99 ± 1.80, 29.29 ± 2.43, 28.24 ± 8.31, and 32.79 ± 1.56%, respectively, compared to VC (Figs. 4A and B).

Interestingly, the results also revealed that all active compounds exhibited a more potent inhibitory effect on WT1 protein expression than their respective crude ethanolic extracts. However, there was no significant difference in the inhibitory effect between the crude ethanolic extracts and their active compounds on CD34 protein levels.

### Effect of crude ethanolic extracts and active compounds on total cell number in KG-1a cells

**Fig. 5:**
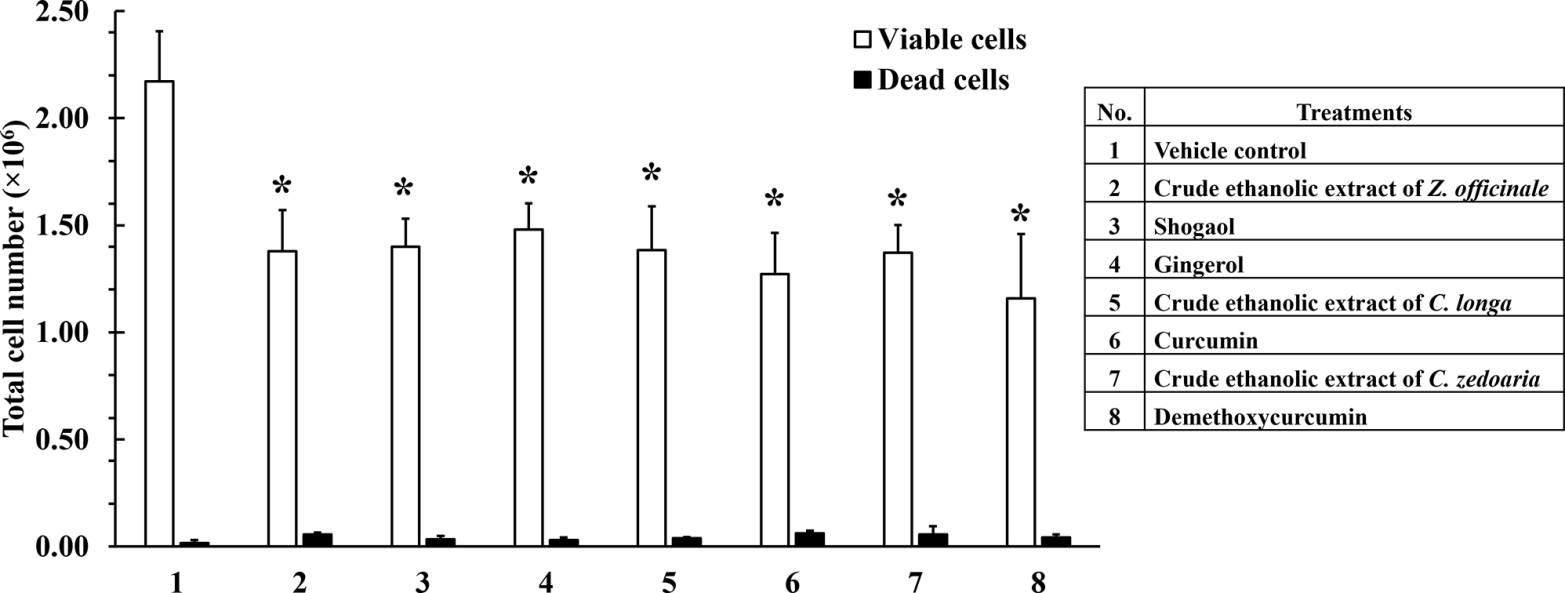
Antiproliferative effects of crude ethanolic extracts and their active compound in KG-1a cells. KG-1a cells were incubated with various concentrations of crude ethanolic extracts and their active compounds for 48 h. Cells were collected and analyzed by trypan blue exclusion method. All treatments significantly decreased the total cell number compared to VC, with no significant difference between crude ethanolic extracts and active compounds. Data are the mean ± SD (*n* = 3); *p < 0.05 vs. VC

The result showed that the total cell number after treatments with crude ethanolic extract of *Z. officinale*, shogaol, gingerol, crude ethanolic extract of *C. longa*, curcumin, crude ethanolic extract of *C. zedoaria*, and bisdemethoxycurcumin with IC_20_ concentrations (Table S9) significantly decreased by 35.95 ± 12.61, 35.32 ± 6.10, 31.39 ± 8.69, 36.32 ± 6.88, 40.75 ± 12.78, 35.97 ± 12.01, and 47.21 ± 7.98%, respectively, when compared to VC. There is no difference in the inhibitory effect between crude ethanolic extracts and their active compounds on total cell numbers (Fig. 5).

To compare the viable and dead cell proportions across treatment groups, normalized cell counts (× 10^3^ cells per 1 million total cells) were calculated. After treatment with the crude ethanolic extracts and their active compounds, the normalized viable cell counts in VC, crude ethanolic extract of *Z. officinale*, shogaol, gingerol, crude ethanolic extract of *C. longa*, curcumin, crude ethanolic extract of *C. zedoaria*, and bisdemethoxycurcumin were 992.15 ± 7.12, 960.41 ± 11.10, 976.13 ± 13.69, 979.50 ± 8.90, 972.17 ± 6.19, 954.40 ± 3.19, 961.98 ± 23.65, and 962.81 ± 19.30 × 10^3^ cells per 1 million total cells, respectively. The normalized dead cell counts in each treatment group were less than 65 × 10^3^ cells per 1 million total cells, with no significant differences in the proportions of viable and dead cells among treatments (Fig. S9).

### Effect of curcumin and shogaol on cell cycle in KG-1a cells

Based on their higher efficacy and SI observed in previous experiments, curcumin and shogaol were prioritized for further evaluation. These compounds significantly reduced WT1 protein expression, supporting their potential as effective anti-leukemic agents. After treatments with various concentrations of active compounds and nocodazole (positive control) in KG-1a cells for 48 h, distinct effects of compounds compared to VC were observed.

**Fig. 6:**
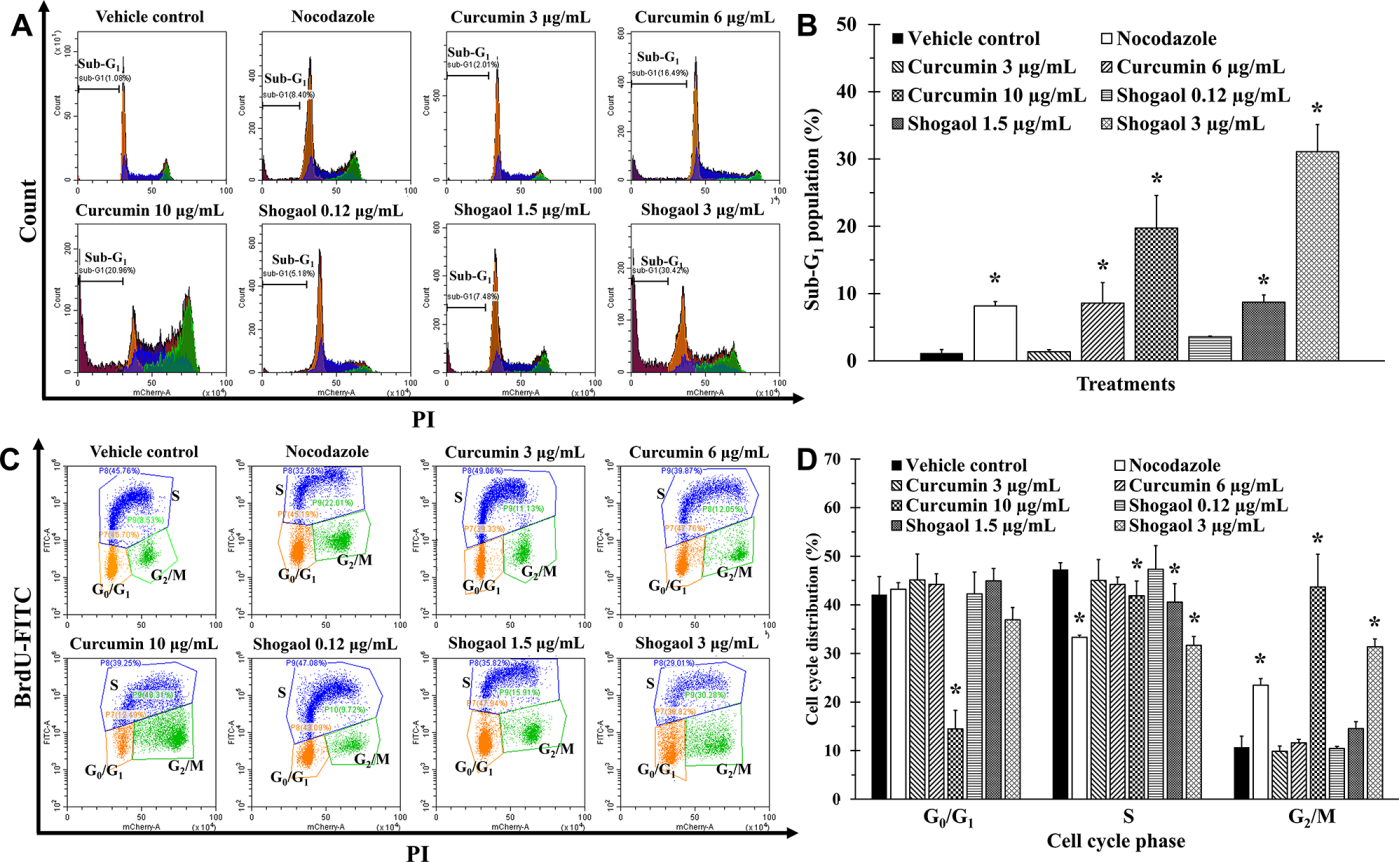
Effect of curcumin, shogaol, and nocodazole on cell cycle distribution in KG-1a cells. Cells were treated with various concentrations of compounds and nocodazole (positive control) for 48 h, and cell cycle distribution was analyzed by flow cytometry. Cell cycle was analyzed by flow cytometry **(A)** Histogram and **(B)** bar graph of the sub-G_1_ population, showing that nocodazole at IC_20_ significantly increased apoptosis. While curcumin and shogaol at IC_20_ did not significantly affect the sub-G_1_ population, both compounds increased apoptosis at higher concentrations. **(C)** Dot plot and **(D)** bar graph of cell cycle distribution, showing that nocodazole at IC_20_ increased the G_2_/M phase population. Curcumin and shogaol did not alter the cell cycle distribution at IC_20_ but increased the G_2_/M phase population and decreased the S phase population at their highest concentrations (IC_50_). Data are the mean ± SD (*n* = 3); *p < 0.05 vs. VC

Apoptosis was evaluated by measuring the sub-G_1_ population using the Nicoletti assay. The results showed that, at its IC_20_ concentration, 6 ng/mL of nocodazole significantly increased the sub-G_1_ population, indicating pronounced apoptosis. In contrast, at their respective IC_20_ concentrations, 3 µg/mL of curcumin and 0.12 µg/mL of shogaol did not significantly increase the sub-G_1_ population compared to VC. However, at higher concentrations, both curcumin and shogaol noticeably induced cell death, with significant increases in the sub-G_1_ population observed (Figs. 6A–B).

To evaluate the effects of curcumin and shogaol on cell cycle arrest, the sub-G_1_ population was excluded, and the distribution of cells across cell cycle phases was analyzed. The results revealed that at the IC_20_ concentration, nocodazole significantly increased the proportion of cells in the G_2_/M phase by 23.48 ± 1.35%, resulting in a significant decrease in the S phase population. In contrast, at their respective IC_20_ concentrations, neither curcumin nor shogaol induced significant changes in the cell cycle phase population compared to VC. However, 1.5 µg/mL of shogaol significantly decreased the S phase population by 40.56 ± 3.82%, although shogaol did not induce cell cycle arrest. At IC_50_ concentrations, both curcumin and shogaol increased the G_2_/M phase population to 43.66 ± 6.75% and 31.38 ± 1.60%, respectively, compared to VC, resulting in a significant decrease in the S phase population (Figs. 6C–D).

### Effect of curcumin and shogaol on cell death in KG-1a cells

**Fig. 7:**
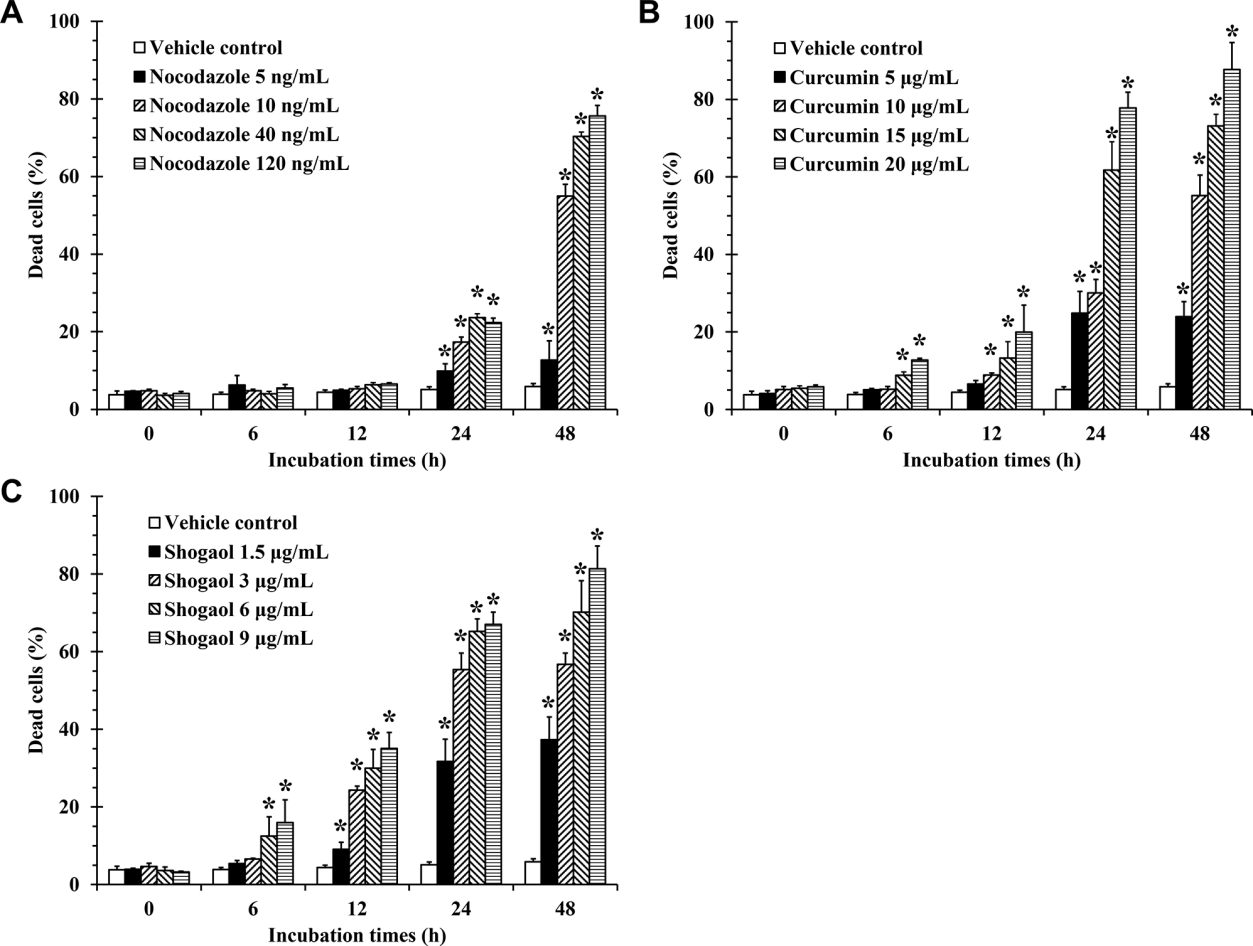
Effect of curcumin, shogaol, and nocodazole on cell death in KG-1a cells. KG-1a cells were incubated with various concentrations of curcumin, shogaol, and nocodazole (positive control) for different incubation times. After collecting the cells, cells were stained with PI solution and analyzed by flow cytometry. Representative bar graph depicting percentage dead cells after treatments with various concentrations and times compared with VC in KG-1a cells. **(A)** Nocodazole induced significant cell death at 24 h, with a dramatic increase at 48 h. **(B)** Curcumin gradually induced cell death, with notable increases after 12 h. **(C)** Shogaol induced cell death, with significant effects at 6 h and further increases over time. Data are the mean ± SD (*n* = 3); *p < 0.05 vs. VC

After KG-1a cells were treated with compounds at their IC_50_ concentrations, the results indicated that both compounds could induce cell death in a dose- and time-dependent manner. Nocodazole (positive control) at concentrations of 5–120 ng/mL showed a significant induction of cell death after 24 h of incubation when compared to VC, and this effect dramatically increased at 48 h of incubation (Fig. 7A). Shogaol at 6–9 µg/mL notably induced cell death at 6 h, and its effect increased continuously across all concentrations with longer incubation times. In contrast, curcumin gradually induced cell death after 12 h of incubation (Figs. 7B and C).

### Effects of curcumin and shogaol on apoptotic related-protein and mechanism of apoptosis in KG-1a cells

To investigate the effects of curcumin and shogaol on apoptotic-related proteins in KG-1a cells, the cells were treated with IC_50_ concentrations of curcumin, shogaol, and nocodazole (positive control) for 0–12 h at 37 °C in a humidified incubator with 5% CO_2_.

**Fig. 8:**
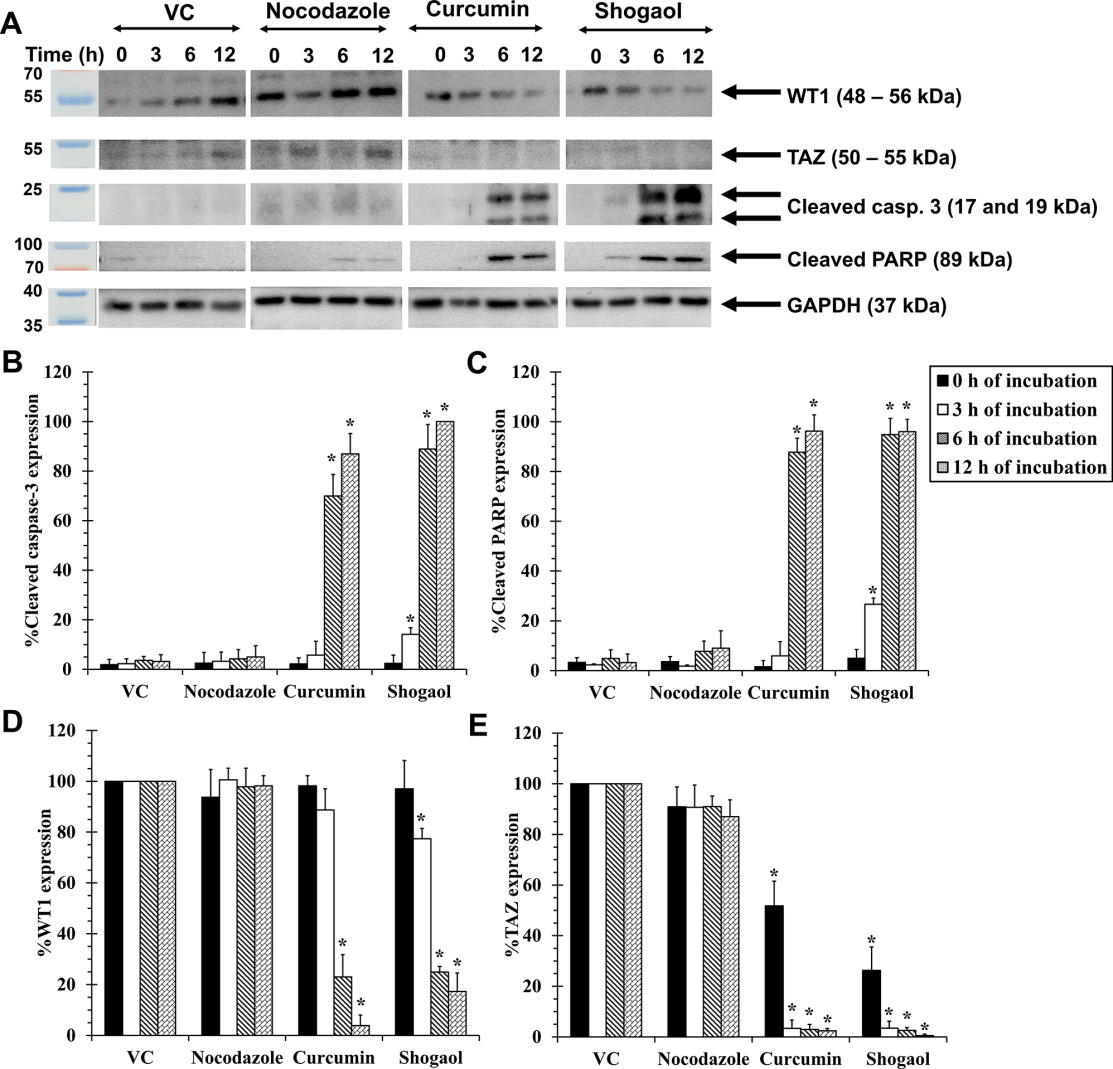
Western blot analysis following curcumin, shogaol, and nocodazole treatments in KG-1a cells. KG-1a cells were incubated with IC_50_ concentration of curcumin, shogaol, and nocodazole (positive control) for 0–12 h. The expression of target proteins was analyzed by Western blotting. (**A**) Protein levels after treatment with the IC_50_ concentrations of curcumin (10 µg/mL), shogaol (3 µg/mL), and nocodazole (10 ng/mL) for 0–12 h. (**B**) Protein expressions for cleaved caspase-3, (**C**) cleaved PARP, (**D**) WT1, and (**E**) TAZ in KG-1a cells after 0–12 h of treatment. The levels of protein were normalized using GAPDH protein levels. Full-length blots are presented in Supplementary file (uncropped gels and blot images, Figs. S17–S21). Shogaol induced the activation of cleaved caspase-3 and PARP at 3 h, while curcumin triggered these proteins at 6 h. WT1 expression significantly decreased with both curcumin and shogaol treatments, but nocodazole had no effect. TAZ levels remained unchanged. Data are the mean ± SD (*n* = 3); *p < 0.05 vs. VC

The results showed that shogaol initiated the activation of cleaved caspase-3 and PARP after 3 h of treatment, whereas curcumin activated both proteins after 6 h. However, nocodazole failed to induce the activation of cleaved caspase-3 and PARP even after 12 h of incubation, compared to VC (Figs. 8A–C).

Furthermore, the levels of WT1 and TAZ protein expressions were investigated using Western blotting after treatments with curcumin and shogaol. The results showed that WT1 protein levels gradually decreased after treatments with curcumin and shogaol compared to non-treatment (0 h incubation time). Notably, the WT1 protein level was significantly decreased by 77.01 ± 8.80% after treatments with curcumin for 6 h, and by 22.58 ± 3.99% after treatment with shogaol for 3 h when compared to VC. Regarding the TAZ protein, no significant change in its abundance was observed. However, nocodazole failed to reduce WT1 expression even after 12 h of incubation (Figs. 8A, D, and E).

After confirming the presence of apoptosis-related proteins, the mechanism of apoptosis induced by these compounds in KG-1a cells was investigated using Western blotting. To explore this mechanism, the expression levels of the phosphorylated forms of c-Jun (p-c-Jun), SAPK/JNK (p-SAPK/JNK), and Akt (p-Akt (S473)) were examined after treating KG-1a cells with curcumin or shogaol for 0–6 h. The results showed that p-c-Jun and p-SAPK/JNK increased after incubation with shogaol or curcumin at 6 h, while p-Akt (S473) decreased at 6 h compared to VC (Fig. S12).

## Discussion

Inhibition of LSCs has been a key focus in leukemia treatment so far [37]. Therefore, LSC targeting therapy is a novel strategy aimed at improving patient’s quality of life after treatments [38, 39]. In this study, KG-1a cell line was used as an LSC model because it exhibits characteristics similar to those of LSCs, including differentiation, proliferation, and self-renewal [40–42]. Plants from the Zingiberaceae family are widely used in traditional medicine across Southeast Asia due to their accessibility and safety. The exploration of the bioactivities of plants in this family holds promise for drug discovery, particularly as potential anticancer agents. Among the 10 crude ethanolic extracts obtained from the rhizomes of Zingiberaceae plants, each extract exhibited varying cytotoxicity across different cancers and leukemic cell lines. Notably, extracts from *C. longa*, *C. zedoaria*, and *Z. officinale* demonstrated significant cytotoxicity against KG-1a leukemic stem cells. Moreover, these extracts exhibited strong cytotoxic effects on K562, MCF-7, and HeLa cells, with less impact on PBMCs. Therefore, *C. longa*, *C. zedoaria*, and *Z. officinale* were selected as the candidate plants for this study. The active compounds of *C. longa* and *C. zedoaria* are curcuminoids. Curcumin is the main compound found in *C. longa*, whereas demethoxycurcumin is the most abundant in *C. zedoaria* [43–45]. *Z. officinale* contains active compounds, namely shogaol and gingerol [46]. The anticancer activities of these active compounds and their crude ethanolic extracts were compared in this study. In the cytotoxicity studies, SI provides insight into the relative safety and efficacy of a compound, assessing its ability to selectively target and impact cancer cells without causing significant harm to normal cells. Values higher than 1 indicate favorable selectivity against cancer cells [35, 36]. In this study, all active compounds exhibited SI values greater than 1, except bisdemethoxycurcumin. Notably, shogaol showed an SI value of 3, indicating its specific targeting ability against KG-1a cells.

Suppressing unregulated inflammatory cytokines (IL-2 and TNF-α) and NO productions from macrophages is one method that can reduce the risk of cancer initiation. In this study, the cancer prevention activities of the active compounds were compared with those of their crude ethanolic extracts. Regarding *C. longa* and *Z. zedoaria* plants, curcuminoids and their crude ethanolic extracts showed no difference in activity. Both compounds decreased the production of IL-2 and TNF-α, which are important inflammatory cytokines as well as NO. Regarding *Z. officinale*, shogaol demonstrated better inhibitory activity against the inflammatory cytokines and NO than either the crude ethanolic extract and gingerol. Therefore, curcuminoids and shogaol emerge as promising compounds for cancer prevention studies.

Previous studies have reported that shogaol, gingerol, and curcuminoids exhibited notable anticancer activity and cancer prevention property. The content of shogaol in the crude ethanolic extract of *Z. officinale* was reported to be very low, constituting less than 2% in fresh rhizome [46]. In the current study, shogaol exhibited significant anticancer activity when compared to gingerol and its crude ethanolic extract. Meanwhile the total curcuminoids content in the crude ethanolic extracts of *C. longa* and *C. zedoaria* is approximately 6% [43, 44]. However, the results indicated no significant difference in cancer prevention and anticancer activities between curcuminoids and their crude ethanolic extracts.

After confirming the anticancer activity of the extracts, we further examined the active compounds to investigate their mechanisms of action and clarify their contributions to the overall activity. However, bisdemethoxycurcumin was excluded from further assays due to its low SI, indicating limited specificity toward KG-1a cells and reduced therapeutic potential.

The WT1 protein is a hallmark in leukemia and plays an integral role in leukemogenesis. Increased WT1 protein expression associated with high rates of cell proliferation [47]. In this study, the effect of active compounds compared to their crude ethanolic extracts on WT1 and CD34 expressions in KG-1a cells were investigated. The result showed that at non-cytotoxic doses, all active compounds and their crude ethanolic extracts significantly decreased WT1 protein expression in KG-1a cells, thereby reducing leukemic cell stem cell proliferation. The normalized viable cell counts revealed a consistent decrease in leukemic cell viability across treatments, aligning with the observed WT1 downregulation. This supports the role of WT1 in maintaining leukemic cell proliferation and suggests that the treatments effectively targeted this pathway. Interestingly, each crude ethanolic extract showed a stronger inhibitory effect on WT1 expression than its active compounds. Notably, the crude ethanolic extract of *Z. officinale* demonstrated greater WT1 downregulation than shogaol, even though shogaol exhibited better cancer prevention properties. This suggests that shogaol may possibly target other leukemia-related proteins apart from WT1. In this study, CD34, a hallmark of the leukemic stem cells that can be phosphorylated by protein kinase C (PKC), was investigated. The results showed that, although all extracts and active compounds could significantly decreased CD34 expression, the inhibitory effects of each treatment were not significantly different.

Despite extensive research on WT1, few studies have explored the Hippo signaling pathway in leukemia. Previous reports have indicated that YAP protein expression in K562 and HL-60 cell lines plays an important role in cell proliferation in leukemic cells [27, 28]. In contrast, our study revealed that KG-1a leukemic stem cells and other leukemic cell lines (K562 and EoL-1) did not express YAP protein, while TAZ was expressed at a low level in KG-1a cells (Figs. S13). However, no significant changes in TAZ expression were observed upon extract treatments. Although YAP/TAZ have been shown to play important roles in chemoresistance in solid tumors, they may not be as relevant in leukemic stem cell chemoresistance.

Since curcumin and shogaol demonstrated higher efficacy and SI in cytotoxicity studies, both compounds were prioritized for further investigation. They significantly inhibited WT1 protein expression, suggesting their strong potential as anti-leukemic agents. Previous reports have indicated that curcuminoids, particularly curcumin, exhibited antiproliferative effects in various leukemic cell lines and patients’ leukemic cells [48, 49]. Moreover, previous studies have shown that curcumin treatment in K562 cells leads to the attenuation of WT1 auto-regulatory function. This occurs through the inhibition of PKCα and c-Jun N-terminal kinase (JNK) signaling, which suppresses both WT1 auto-regulation and c-Jun/AP-1 binding to its cognate consensus site at the proximal *WT1* gene promoter [50, 51]. Furthermore, curcumin decreased cell proliferation by reducing WT1 protein levels in KG-1a stem cell lines [32]. Meanwhile, 6-shogaol from *Z. officinale* played a major role in inducing apoptosis in acute lymphoblastic leukemia (ALL) by activating p53 and generating oxidative stress [52]. Additionally, shogaol suppressed proliferation in oral squamous cell carcinoma (OSCC) cells and induced apoptosis by inhibiting the phosphatidylinositol 3-kinase (PI3K)/Akt signaling pathway, while regulating apoptosis-associated factors such as p53, Bax, Bcl-2, and cleaved caspase-3 [53]. This study is the first to report that shogaol can inhibit WT1 and CD34 expressions.

The PI3K/Akt signaling pathway and JNK pathway regulate numerous cellular processes, including cell growth, migration, invasion, and apoptosis. Phosphorylation of Akt enhances CDK2 activity, which is required for cell progression from the S to G_2_/M phase and inhibits apoptosis [54–56]. Therefore, the inhibition of PI3K/Akt can induce caspase-dependent apoptosis [55, 57]. In this study, curcumin and shogaol were selected for further investigation due to their efficacy as active compounds from their respective candidate plants. While both curcumin and shogaol demonstrated the ability to decrease WT1 protein levels and inhibit cell proliferation at the IC_20_ concentrations, non-cytotoxic doses were insufficient to arrest the cell cycle in KG-1a cells. IC_50_ concentrations were required to induce cell cycle arrest at the G_2_/M phase and promote cell death. Moreover, time-course experiments indicated that both compounds could induce cell death in a dose- and time-dependent manner. To explore the mechanism of curcumin and shogaol in inducing cell death in KG-1a cells, the cells were treated with the IC_50_ concentration of each compound. The results indicated that both curcumin and shogaol induced apoptosis by decreasing the phosphorylation of Akt (S473), leading to an increase in the levels of phosphorylated JNK and c-Jun. Subsequently, this process resulted in an upregulation of cleaved caspase-3 and PARP proteins. Additionally, both curcumin and shogaol decreased the expression of WT1 and TAZ proteins, which are associated with leukemic cell proliferation.

## Conclusion

Our findings suggest that *C. longa*, *C. zedoaria*, and *Z. officinale* extracts are potential candidates for cancer treatment, exhibiting significant cytotoxicity across various cancer cell types. Additionally, these extracts and their active compounds demonstrate cancer prevention properties by inhibiting the production of IL-2, TNF-α, and NO. Furthermore, curcumin and shogaol can arrest the cell cycle and induce apoptosis in KG-1a cells through the inhibition of the Akt pathway. Thus, curcumin and shogaol emerge as promising compounds for leukemia treatment, requiring further investigation.

## Electronic supplementary material

Below is the link to the electronic supplementary material.

Supplementary Material 1

Supplementary Material 2

## Data Availability

The datasets used and/or analyzed during the current study are available from the corresponding author by reasonable request.

## Abbreviations

ALL: Acute lymphoblastic leukemia cells
AML: Acute myeloid leukemia
CDK2: Cyclin-dependent kinase 2
CML: Chronic myeloid leukemia
DMEM: Dulbecco’s Modified Eagle Medium
HSCs: Hematopoietic stem cells
IC_20_: Non-cytotoxic dose (Inhibitory concentration values at 20% growth)
IC_50_: Inhibitory concentration values at 50% growth
IL-2: Interleukin-2
IMDM: Iscove’s Modified Dulbecco’s Medium
JNK: c-Jun N-terminal kinase
LPS: Lipopolysaccharide
LSCs: Leukemic stem cells
MRD: Minimal residual disease
NCI: National Cancer Institute
NO: Nitric oxide
OD: Optical density
OSCC: Oral squamous cell carcinoma
p-c-Jun: Phosphorylated forms of c-Jun
p-SAPK/JNK: Phosphorylated forms of SAPK/JNK
p-Akt: Phosphorylated form of Akt
PBMCs: Peripheral blood mononuclear cells
PBS: Phosphate buffer saline
PBS-T: Phosphate buffer saline with Tween-20
PHA: Phytohemagglutinin
PI: Propidium iodide
PI3K: Phosphatidylinositol 3-kinase
PKC: Protein kinase C
RPMI-1640: Roswell Park Memorial Institute-1640
RT: Room temperature
SD: Standard derivation
SI: Selectivity index
TNF-α: Tumor necrosis factor-alpha
VC: Vehicle control
WT1: Wilms’ tumor 1
