# Supplementary material for: Anticancer and cancer preventive activities of shogaol and curcumin from Zingiberaceae family plants in KG-1a leukemic stem cells
Source: BMC Complement Med Ther. 2025 Feb 28;25:87. doi: 10.1186/s12906-025-04829-7 (PMC11869560; doi:10.1186/s12906-025-04829-7)
Supplement: Supplementary file 1 — Supplementary Material 1 [file 12906_2025_4829_MOESM1_ESM.pdf]

Supplementary data

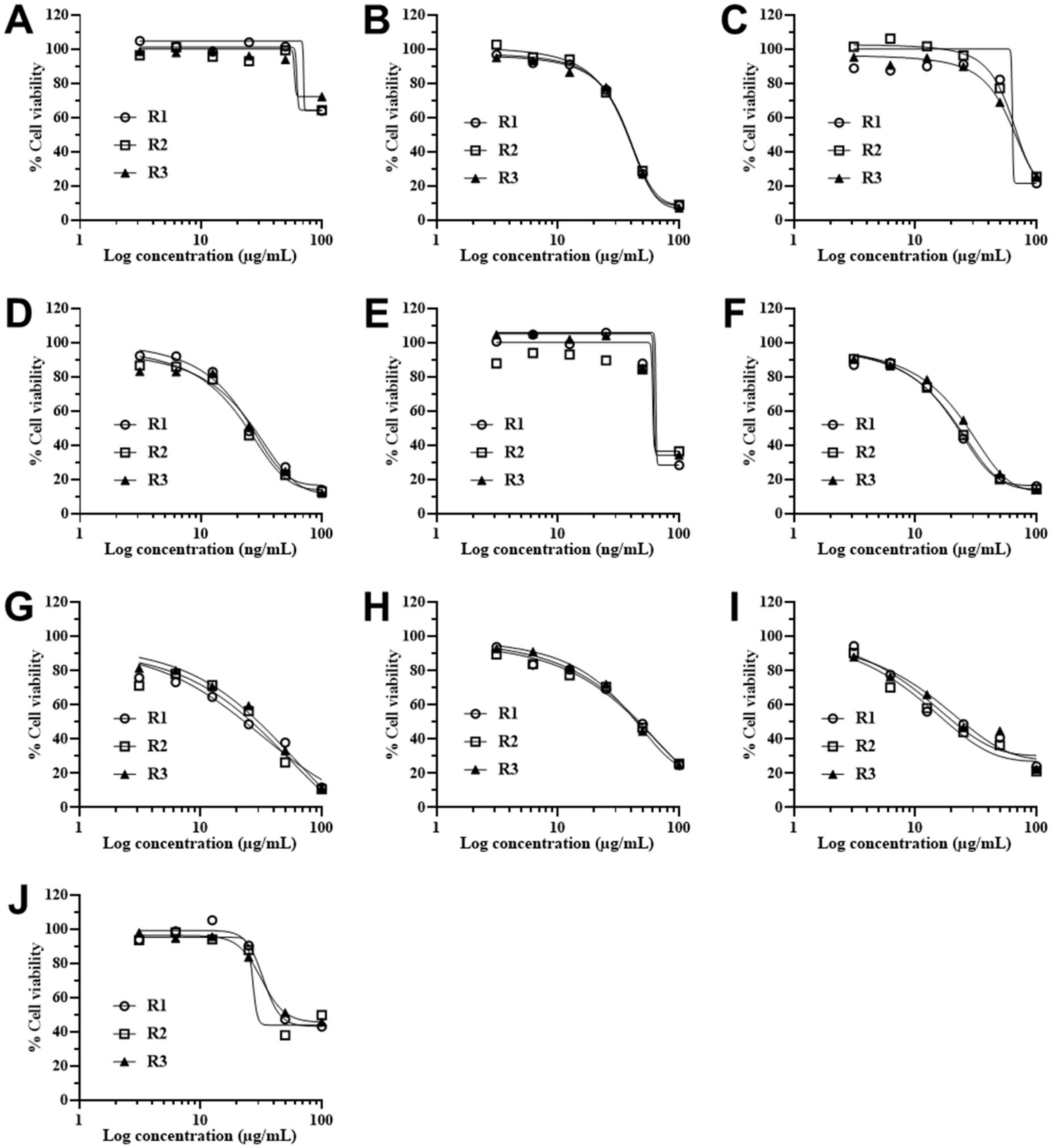

Fig. S1 Non-linear regression graphs from the MTT assay used for IC_{50} determination of cytotoxicity in KG-1a cells treated with crude ethanolic extracts of (A) *A. galanga*, (B) *B. rotunda*, (C) *C. aeruginosa*, (D) *C. longa*, (E) *C. mangga*, (F) *C. zedoaria*, (G) *K. parviflora*, (H) *Z. montanum*, (I) *Z. officinale*, and (J) *Z. ottensii*. Each line represents a replicate: R1 = first replicate, R2 = second replicate, and R3 = third replicate.

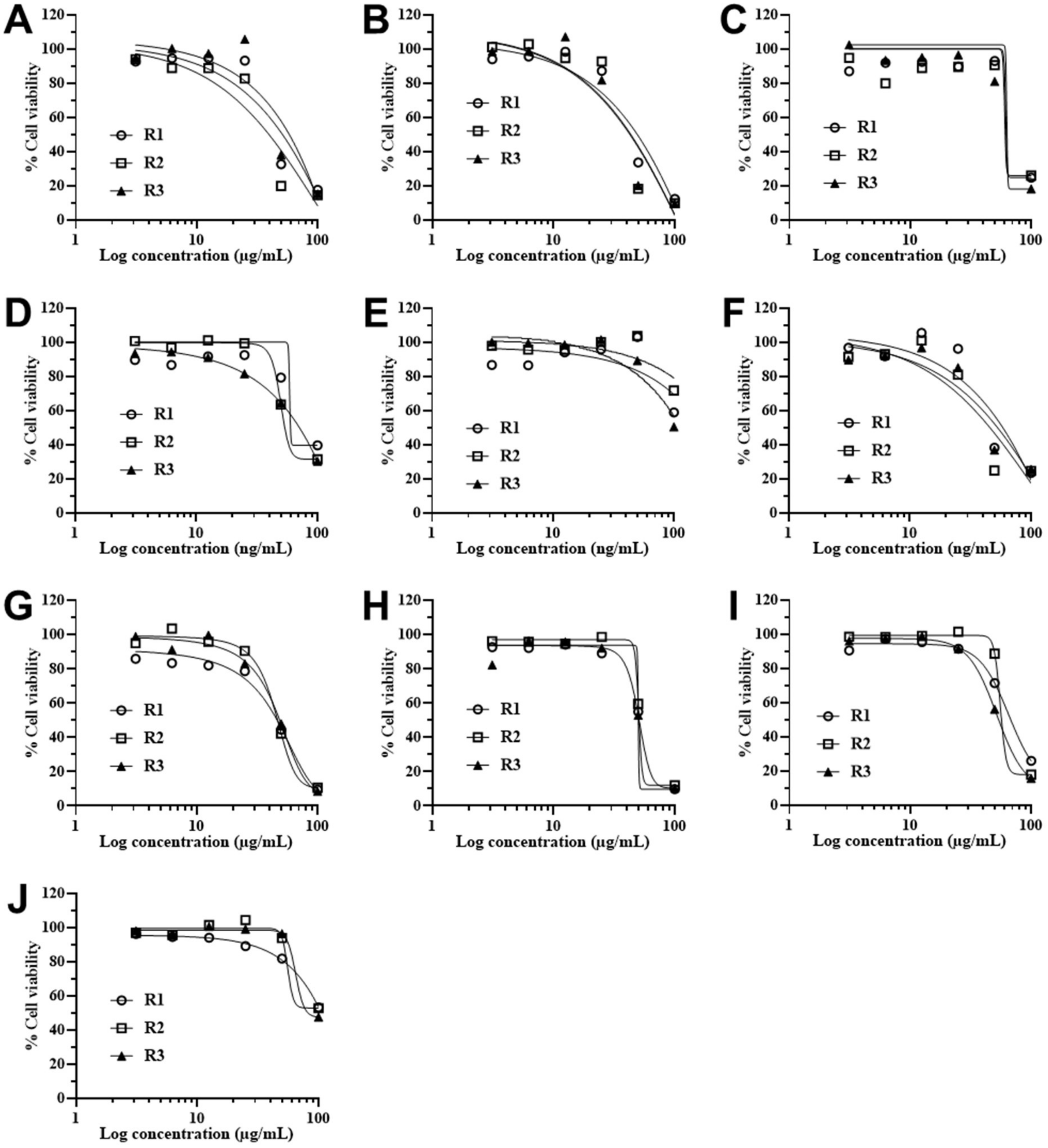

Fig. S2 Non-linear regression graphs from the MTT assay used for IC_{50} determination of cytotoxicity in PBMCs treated with crude ethanolic extracts of (A) *A. galanga*, (B) *B. rotunda*, (C) *C. aeruginosa*, (D) *C. longa*, (E) *C. mangga*, (F) *C. zedoaria*, (G) *K. parviflora*, (H) *Z. montanum*, (I) *Z. officinale*, and (J) *Z. ottensii*. Each line represents a replicate: R1 = first replicate, R2 = second replicate, and R3 = third replicate.

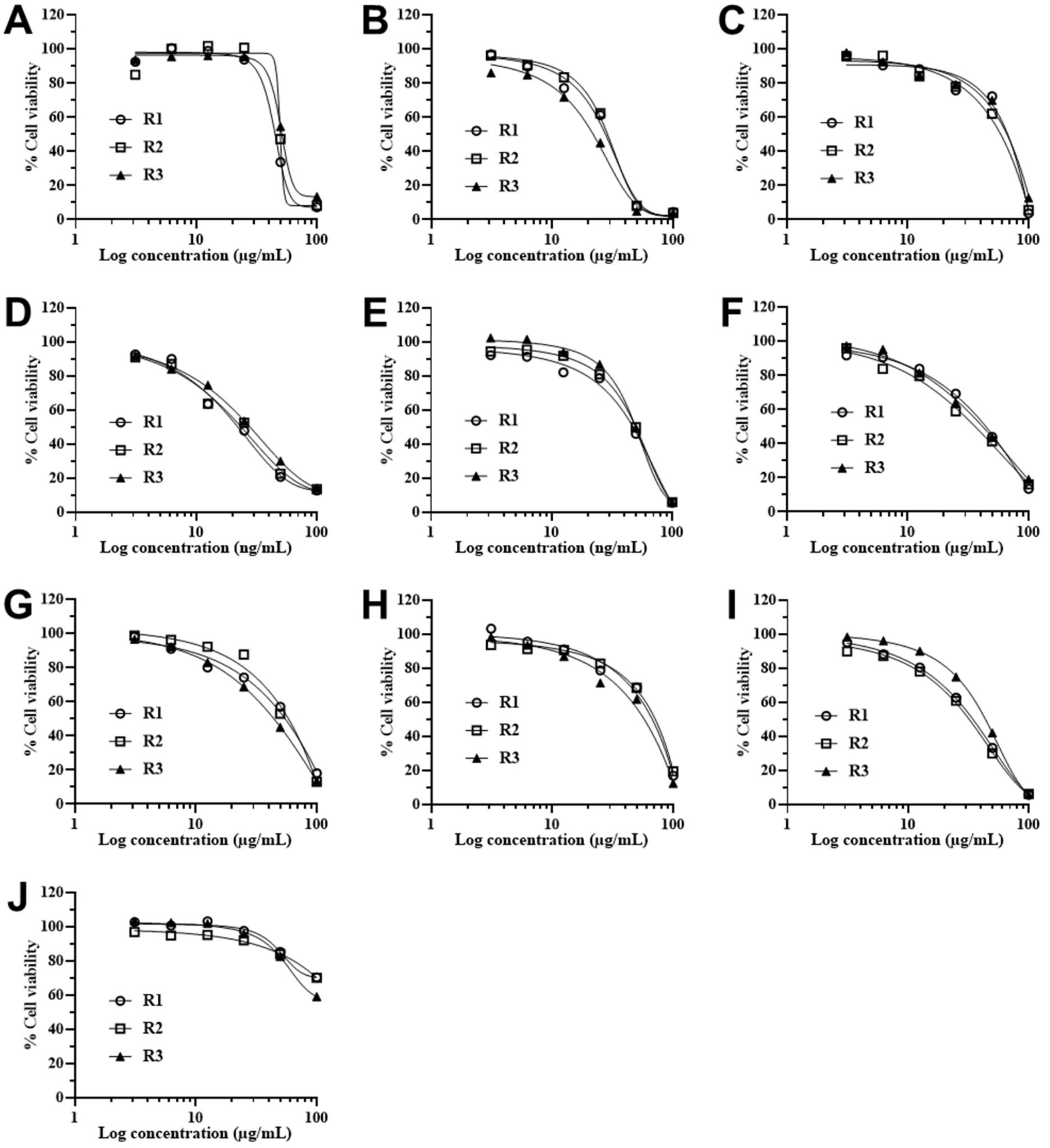

Fig. S3 Non-linear regression graphs from the MTT assay used for IC_{50} determination of cytotoxicity in K562 cells treated with crude ethanolic extracts of (A) *A. galanga*, (B) *B. rotunda*, (C) *C. aeruginosa*, (D) *C. longa*, (E) *C. mangga*, (F) *C. zedoaria*, (G) *K. parviflora*, (H) *Z. montanum*, (I) *Z. officinale*, and (J) *Z. ottensii*. Each line represents a replicate: R1 = first replicate, R2 = second replicate, and R3 = third replicate.represents a replicate: R1 = first replicate, R2 = second replicate, and R3 = third replicate.

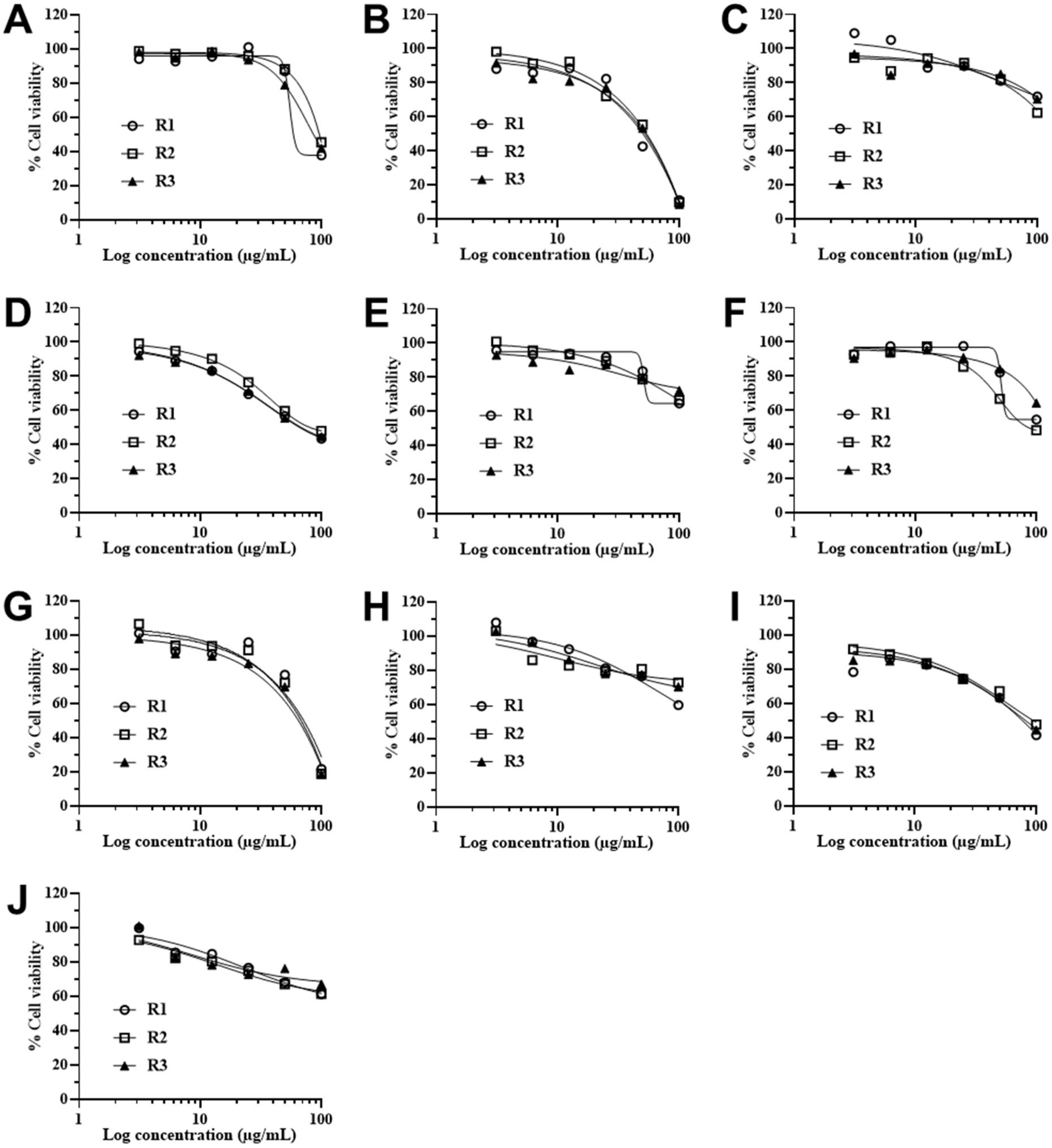

Fig. S4 Non-linear regression graphs from the MTT assay used for IC_{50} determination of cytotoxicity in A549 cells treated with crude ethanolic extracts of (A) *A. galanga*, (B) *B. rotunda*, (C) *C. aeruginosa*, (D) *C. longa*, (E) *C. mangga*, (F) *C. zedoaria*, (G) *K. parviflora*, (H) *Z. montanum*, (I) *Z. officinale*, and (J) *Z. ottensii*. Each line represents a replicate: R1 = first replicate, R2 = second replicate, and R3 = third replicate.

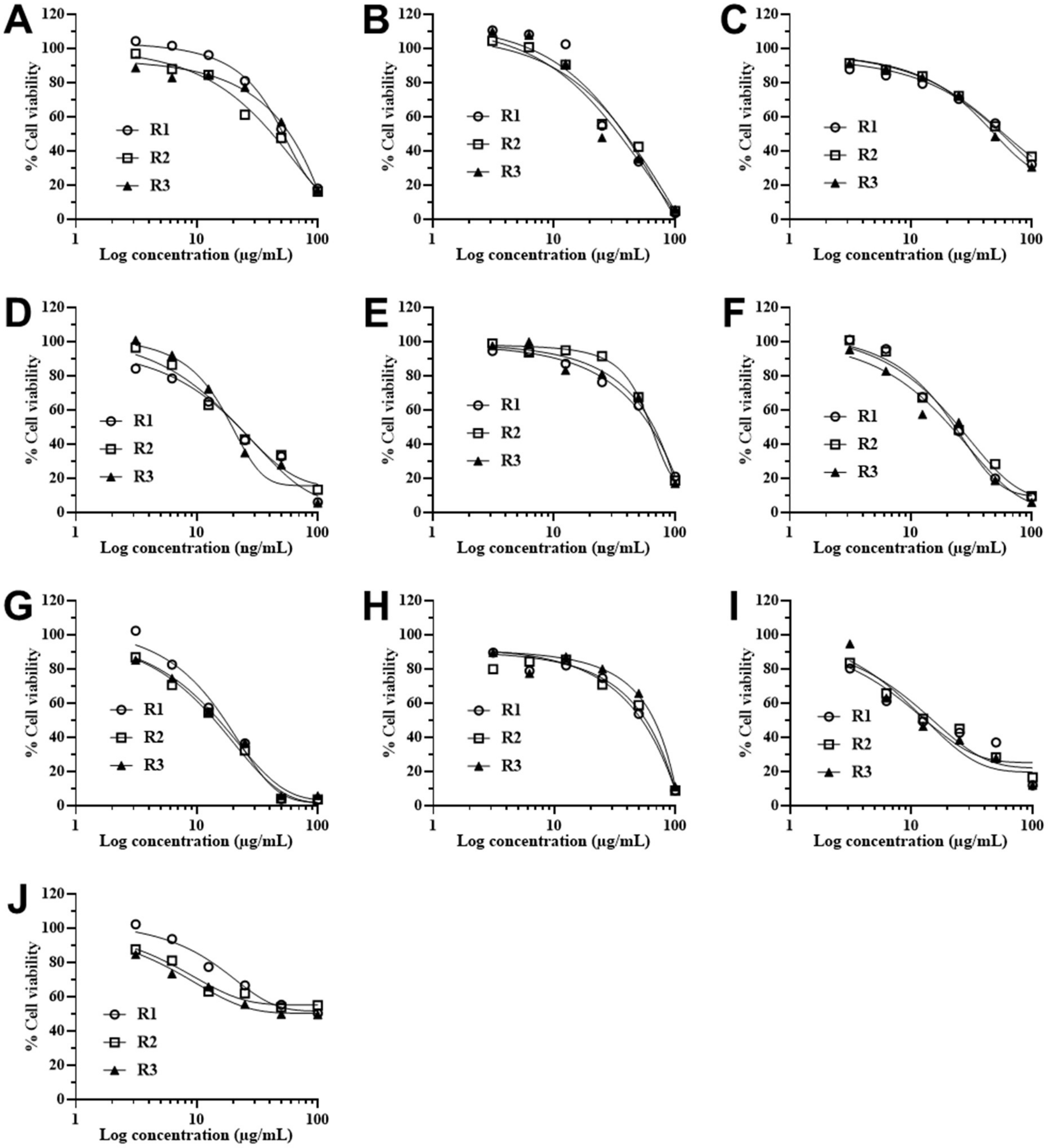

Fig. S5 Non-linear regression graphs from the MTT assay used for IC_{50} determination of cytotoxicity in HeLa cells treated with crude ethanolic extracts of (A) *A. galanga*, (B) *B. rotunda*, (C) *C. aeruginosa*, (D) *C. longa*, (E) *C. mangga*, (F) *C. zedoaria*, (G) *K. parviflora*, (H) *Z. montanum*, (I) *Z. officinale*, and (J) *Z. ottensii*. Each line represents a replicate: R1 = first replicate, R2 = second replicate, and R3 = third replicate.

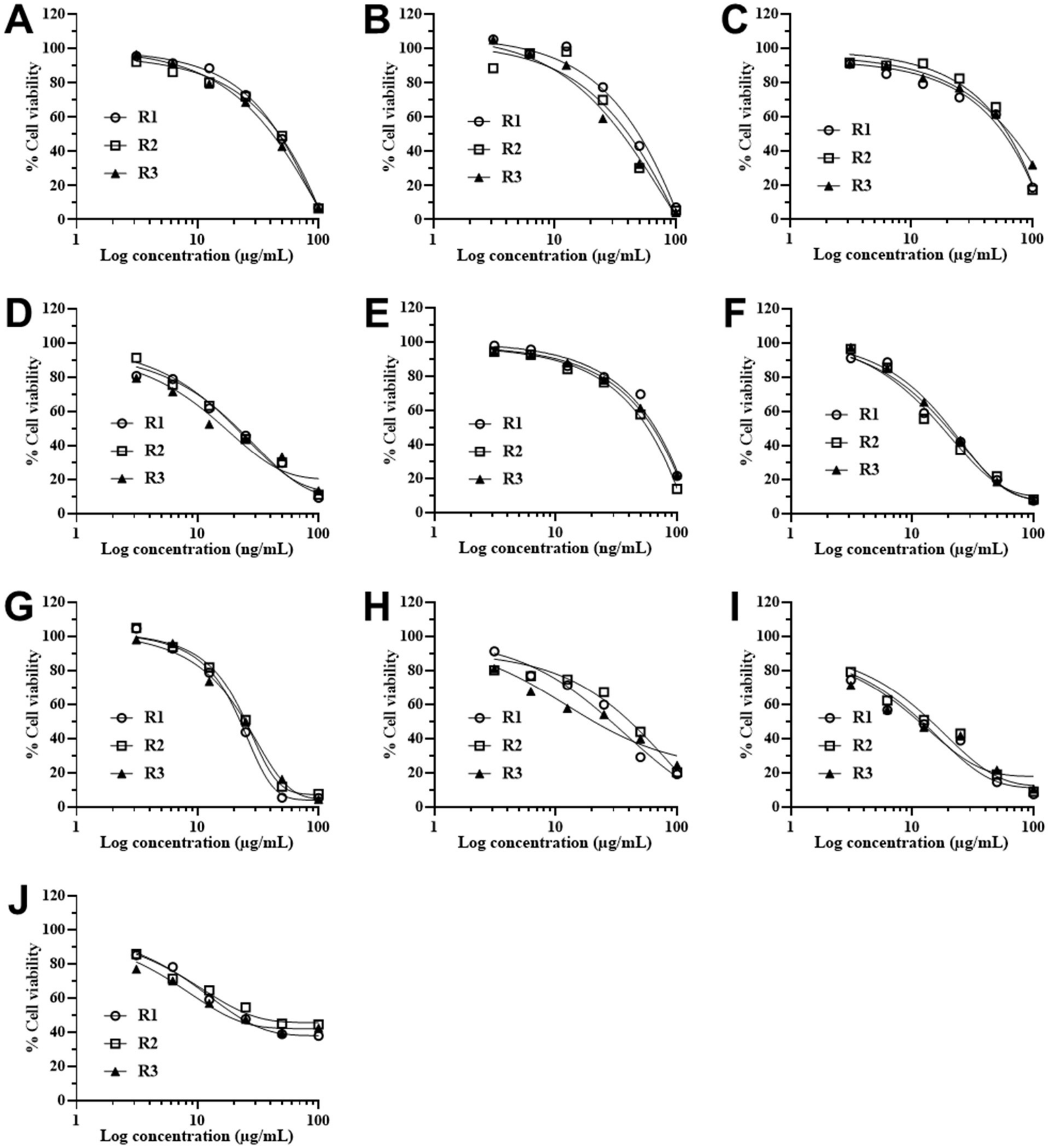

Fig. S6 Non-linear regression graphs from the MTT assay used for IC_{50} determination of cytotoxicity in MCF-7 cells treated with crude ethanolic extracts of (A) *A. galanga*, (B) *B. rotunda*, (C) *C. aeruginosa*, (D) *C. longa*, (E) *C. mangga*, (F) *C. zedoaria*, (G) *K. parviflora*, (H) *Z. montanum*, (I) *Z. officinale*, and (J) *Z. ottensii*. Each line represents a replicate: R1 = first replicate, R2 = second replicate, and R3 = third replicate.

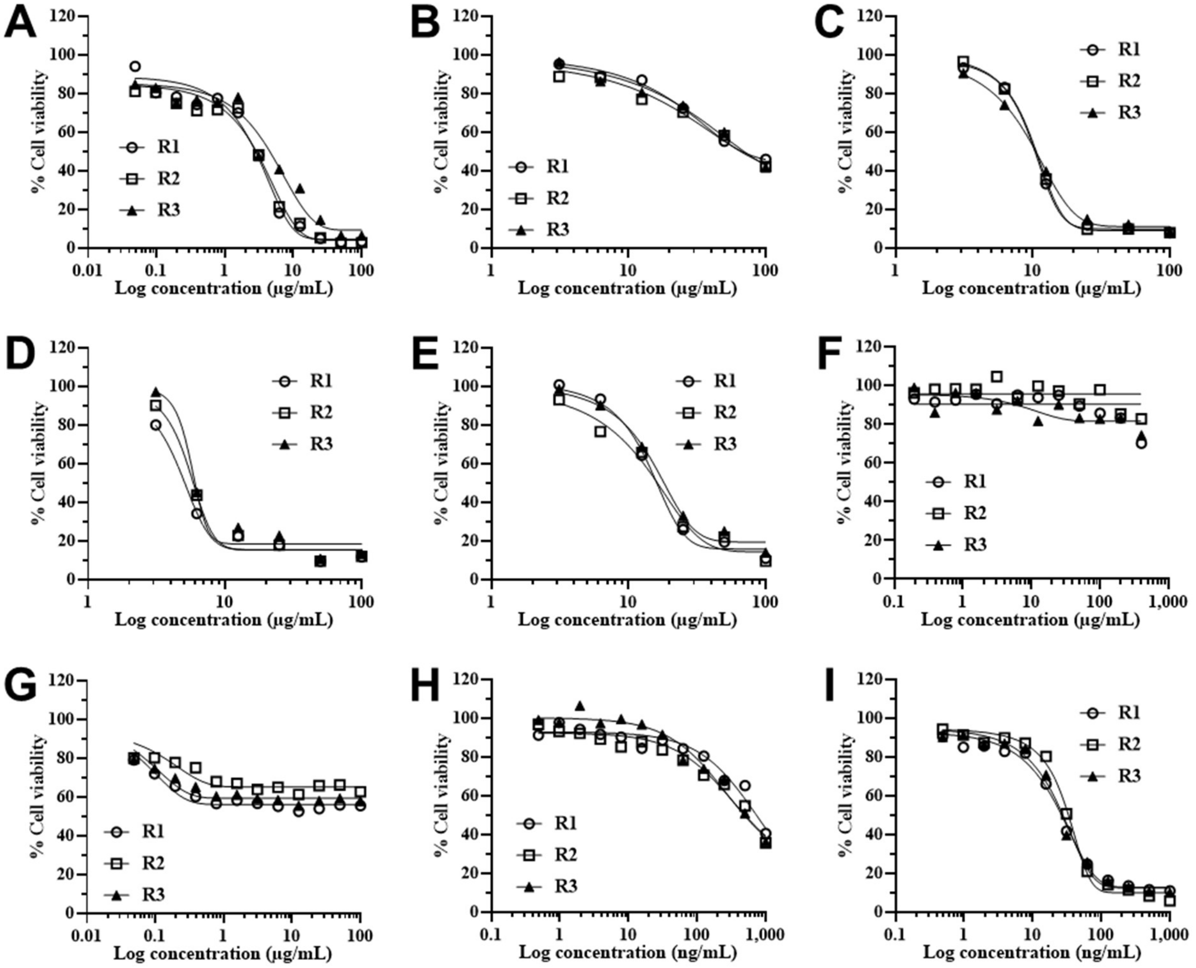

Fig. S7 Non-linear regression graphs from the MTT assay used for IC_{50} determination of cytotoxicity in KG-1a cells treated with (A) shogaol, (B) gingerol, (C) curcumin, (D) demethoxycurcumin, (E) bisdemethoxycurcumin, (F) cyclophosphamide, (G) cytarabine, (H) doxorubicin, and (I) idarubicin. Each line represents a replicate: R1 = first replicate, R2 = second replicate, and R3 = third replicate.

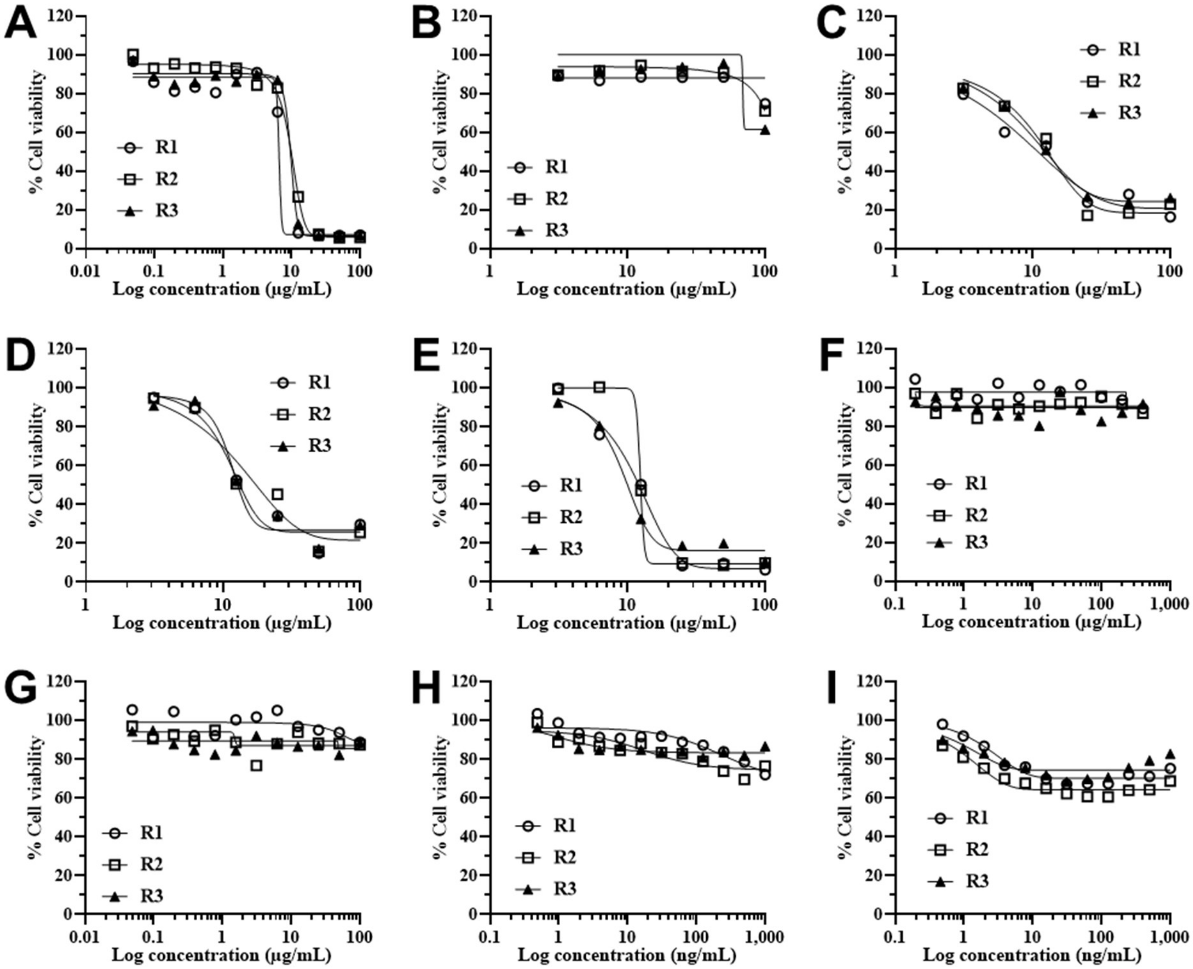

Fig. S8 Non-linear regression graphs from the MTT assay used for IC_{50} determination of cytotoxicity in PBMCs treated with (A) shogaol, (B) gingerol, (C) curcumin, (D) demethoxycurcumin, (E) bisdemethoxycurcumin, (F) cyclophosphamide, (G) cytarabine, (H) doxorubicin, and (I) idarubicin. Each line represents a replicate: R1 = first replicate, R2 = second replicate, and R3 = third replicate.

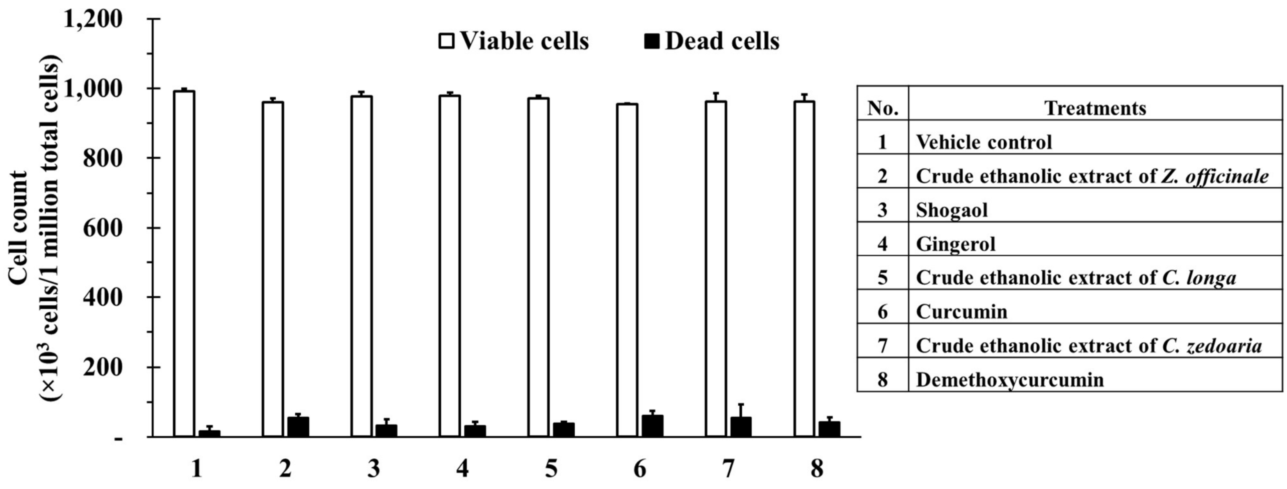

No.	Treatments
1	Vehicle control
2	Crude ethanolic extract of *Z. officinale*
3	Shogaol
4	Gingerol
5	Crude ethanolic extract of *C. longa*
6	Curcumin
7	Crude ethanolic extract of *C. zedoaria*
8	Demethoxycurcumin

Fig. S9 Normalized viable and dead cell counts (×10^3 cells per 1 million total cells) in KG-1a cells treated with crude ethanolic extracts and their active compounds for 48 h. Cells were collected and analyzed using trypan blue exclusion method. Viable cell counts (white bars) and dead cell counts (black bars) were normalized to a total of 1 million cells per treatment group. Data are mean ± SD (n = 3).

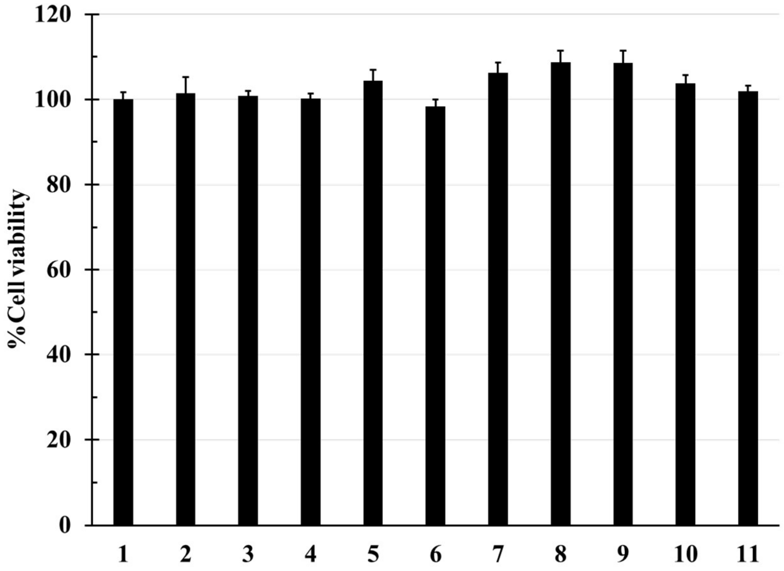

No.	Treatments
1	Vehicle control
2	Lipopolysaccharide
3	Dexamethasone
4	Crude ethanolic extract of *Z. officinale*
5	Shogaol
6	Gingerol
7	Crude ethanolic extract of *C. longa*
8	Crude ethanolic extract of *C. zedoaria*
9	Curcumin
10	Demethoxycurcumin
11	Bisdemethoxycurcumin

Fig. S10 Cell viability of RAW264.7 cells after treatment with crude ethanolic extracts and their active compound for 24 h. Data are the mean ± SD (n = 3).

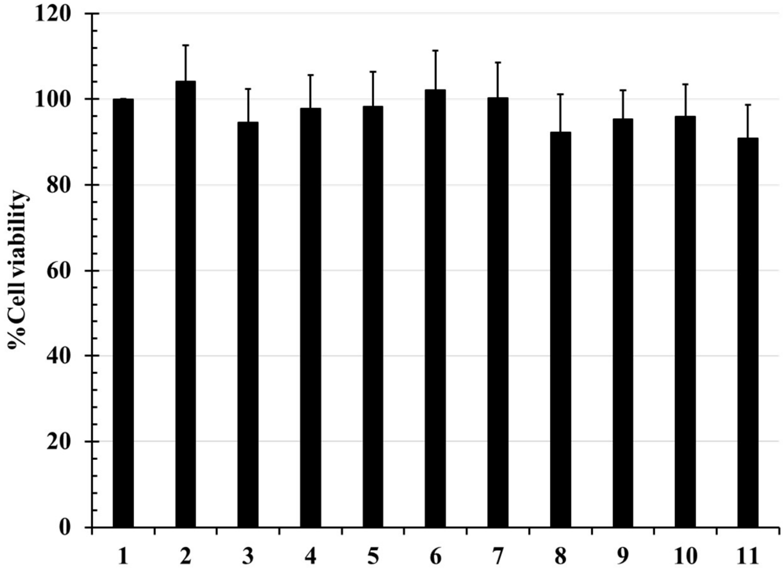

No.	Treatments
1	Vehicle control
2	Phytohemagglutinin
3	Dexamethasone
4	Crude ethanolic extract of *Z. officinale*
5	Shogaol
6	Gingerol
7	Crude ethanolic extract of *C. longa*
8	Crude ethanolic extract of *C. zedoaria*
9	Curcumin
10	Demethoxycurcumin
11	Bisdemethoxycurcumin

Fig. S11 Cell viability of PBMCs after treatment with crude ethanolic extracts and their active compound for 24 h. Data are the mean ± SD (n = 3).

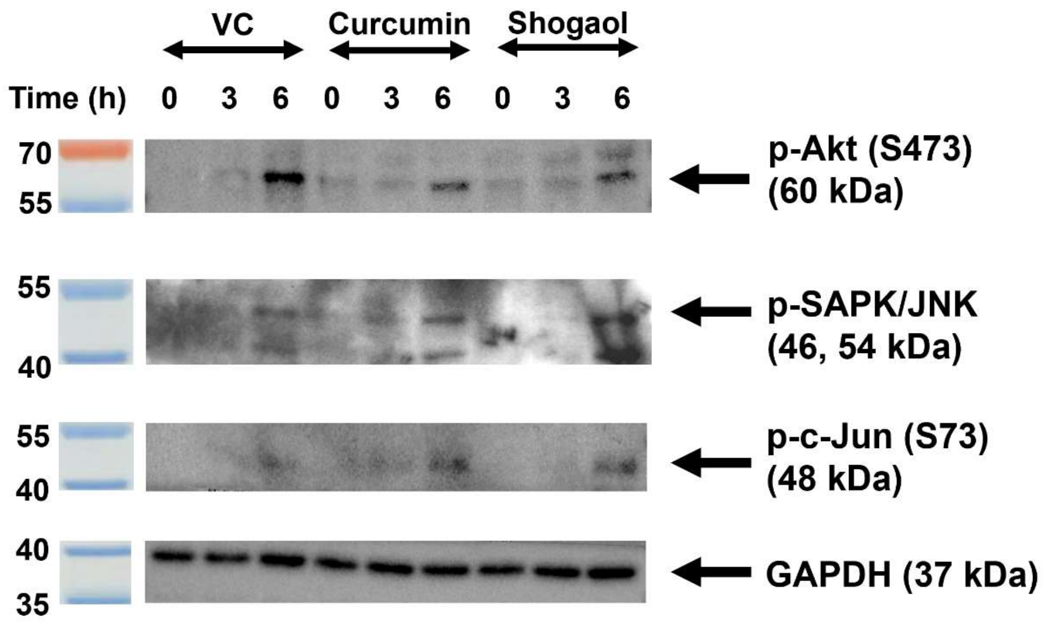

Fig. S12 Western blot analysis of apoptosis mechanism after treatment with IC_{50} concentration of curcumin and shogaol compared with vehicle control in KG-1a cells. Full-length blots are presented in Additional data (Figs. S22–S25).

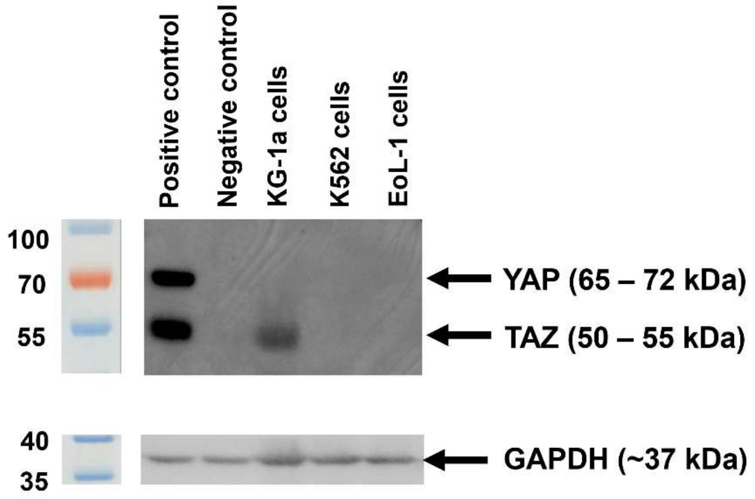

Fig. S13 Western blot analysis of YAP/TAZ protein expression in three leukemic cell lines compared with control. Positive control = wild-type Lewis lung carcinoma cells and negative control = YAP/TAZ knock-out Lewis lung carcinoma cells. Full-length blots are presented in Additional data (Figs. S26–S27).

Table S1 Cytotoxicity of crude ethanolic extracts against KG-1a cells by MTT assay.

Crude ethanolic extracts	IC_{50} value (μg/mL)				
	**R1**	**R2**	**R3**	**Mean**	**SD**
A. galanga	>100	>100	>100	>100	-
B. rotunda	38.29	38.42	38.67	38.46	0.20
C. aeruginosa	76.49	76.13	71.78	74.80	2.62
C. longa	24.34	23.33	26.10	24.59	1.40
C. mangga	81.73	85.72	84.45	83.97	2.04
C. zedoaria	22.43	23.12	28.61	24.72	3.39
K. parviflora	23.86	30.23	33.94	29.34	5.09
Z. montanum	48.57	46.42	44.95	46.65	1.82
Z. officinale	22.72	19.58	22.79	21.70	1.83
Z. ottensii	48.35	43.91	59.70	50.66	8.14

R1 = first replicate, R2 = second replicate, and R3 = third replicate

Table S2 Cytotoxicity of crude ethanolic extracts against PBMCs by MTT assay.

Crude ethanolic extracts	IC_{50} value (μg/mL)				
	**R1**	**R2**	**R3**	**Mean**	**SD**
A. galanga	42.79	37.98	45.56	42.11	3.84
B. rotunda	42.32	39.31	37.86	39.83	2.27
C. aeruginosa	81.43	81.30	74.59	79.11	3.92
C. longa	86.84	71.00	71.06	76.30	9.13
C. mangga	>100	>100	>100	>100	-
C. zedoaria	44.89	38.77	43.13	42.26	3.15
K. parviflora	45.91	45.91	48.48	46.77	1.48
Z. montanum	55.45	59.99	53.32	56.25	3.41
Z. officinale	73.44	77.23	57.46	69.38	10.50
Z. ottensii	>100	>100	>100	>100	-

R1 = first replicate, R2 = second replicate, and R3 = third replicate

Table S3 Cytotoxicity of crude ethanolic extracts against K562 cells by MTT assay.

Crude ethanolic extracts	IC_{50} value (µg/mL)				
	**R1**	**R2**	**R3**	**Mean**	**SD**
A. galanga	43.20	48.68	55.43	49.10	6.13
B. rotunda	30.19	30.64	22.66	27.83	4.49
C. aeruginosa	66.16	60.57	67.38	64.70	3.63
C. longa	23.26	27.11	28.61	26.32	2.76
C. mangga	46.92	49.78	49.52	48.74	1.58
C. zedoaria	43.90	37.47	42.15	41.17	3.33
K. parviflora	58.91	53.52	44.70	52.38	7.18
Z. montanum	67.91	68.73	61.79	66.14	3.79
Z. officinale	35.72	33.68	43.88	37.76	5.40
Z. ottensii	>100	>100	>100	>100	-

R1 = first replicate, R2 = second replicate, and R3 = third replicate

Table S4 Cytotoxicity of crude ethanolic extracts against A549 cells by MTT assay.

Crude ethanolic extracts	IC_{50} value (µg/mL)				
	**R1**	**R2**	**R3**	**Mean**	**SD**
A. galanga	87.41	94.49	88.82	90.24	3.75
B. rotunda	45.32	55.79	53.67	51.60	5.54
C. aeruginosa	>100	>100	>100	>100	-
C. longa	74.83	90.75	74.71	80.10	9.23
C. mangga	>100	>100	>100	>100	-
C. zedoaria	>100	>100	>100	>100	-
K. parviflora	74.18	70.64	69.18	71.33	2.57
Z. montanum	>100	>100	>100	>100	-
Z. officinale	80.17	93.66	85.27	86.37	6.81
Z. ottensii	>100	>100	>100	>100	-

R1 = first replicate, R2 = second replicate, and R3 = third replicate

Table S5 Cytotoxicity of crude ethanolic extracts against HeLa cells by MTT assay.

Crude ethanolic extracts	IC_{50} value (μg/mL)				
	R1	R2	R3	Mean	SD
A. galanga	53.82	45.29	58.42	52.51	6.66
B. rotunda	30.56	35.35	24.33	30.08	5.52
C. aeruginosa	62.66	61.91	48.16	57.57	8.16
C. longa	20.66	20.46	19.90	20.34	0.39
C. mangga	64.95	67.77	66.92	66.55	1.45
C. zedoaria	23.41	23.76	26.88	24.68	1.91
K. parviflora	16.85	14.91	15.88	15.88	0.97
Z. montanum	53.93	58.61	64.21	58.92	5.15
Z. officinale	12.06	14.00	11.08	12.38	1.49
Z. ottensii	>100	>100	>100	>100	-

R1 = first replicate, R2 = second replicate, and R3 = third replicate

Table S6 Cytotoxicity of crude ethanolic extracts against MCF-7 cells by MTT assay.

Crude ethanolic extracts	IC_{50} value (μg/mL)				
	R1	R2	R3	Mean	SD
A. galanga	47.19	48.84	42.98	46.34	3.02
B. rotunda	44.96	37.53	33.55	38.68	5.79
C. aeruginosa	63.15	65.96	69.05	66.05	2.95
C. longa	21.68	20.98	16.03	19.56	3.08
C. mangga	70.13	58.61	64.23	64.32	5.76
C. zedoaria	19.17	16.28	21.06	18.83	2.41
K. parviflora	22.73	25.45	24.81	24.33	1.42
Z. montanum	32.93	43.37	31.84	36.05	6.36
Z. officinale	11.23	13.72	10.38	11.78	1.73
Z. ottensii	22.31	36.32	21.45	26.69	8.35

R1 = first replicate, R2 = second replicate, and R3 = third replicate

Table S7 Cytotoxicity of active compounds and chemotherapeutic drugs against KG-1a cells by MTT assay.

Active compounds and drugs	IC_{50} value				
	**R1**	**R2**	**R3**	**Mean**	**SD**
Shogaol (µg/mL)	2.98	3.00	2.99	2.99	0.01
Gingerol (µg/mL)	77.86	75.36	77.08	76.77	1.28
Curcumin (µg/mL)	10.39	10.57	10.60	10.52	0.12
Demethoxycurcumin (µg/mL)	10.32	11.63	11.89	11.28	0.85
Bisdemethoxycurcumin (µg/mL)	17.13	17.58	19.02	17.91	0.99
Cyclophosphamide (µg/mL)	>400	>400	>400	>400	-
Cytarabine (µg/mL)	>100	>100	>100	>100	-
Doxorubicin (ng/mL)	806.89	622.04	527.61	652.18	142.06
Idarubicin (ng/mL)	25.87	31.79	25.99	27.89	3.38

R1 = first replicate, R2 = second replicate, and R3 = third replicate

Table S8 Cytotoxicity of active compounds and chemotherapeutic drugs against PBMCs by MTT assay.

Active compounds and drugs	IC_{50} value				
	**R1**	**R2**	**R3**	**Mean**	**SD**
Shogaol (µg/mL)	8.30	9.90	9.35	9.18	0.82
Gingerol (µg/mL)	>100	>100	>100	>100	-
Curcumin (µg/mL)	13.77	14.60	12.96	13.78	0.82
Demethoxycurcumin (µg/mL)	14.15	13.59	13.88	13.87	0.28
Bisdemethoxycurcumin (µg/mL)	12.55	12.16	10.21	11.64	1.26
Cyclophosphamide (µg/mL)	>400	>400	>400	>400	-
Cytarabine (µg/mL)	>100	>100	>100	>100	-
Doxorubicin (ng/mL)	>1,000	>1,000	>1,000	>1,000	-
Idarubicin (ng/mL)	>1,000	>1,000	>1,000	>1,000	-

R1 = first replicate, R2 = second replicate, and R3 = third replicate

Table S9 Inhibitory concentration values at 20% growth (IC_{20} values) of crude ethanolic extracts and their active compound on KG-1a, RAW264.7, and PBMCs after incubation for 48 h.

**Compounds**	**IC_{20} (µg/mL)**		
	**KG-1a**	**RAW264.7**	**PBMCs**
Crude ethanolic extract of *Z. officinale*	5.24 ± 0.53	5.94 ± 2.02	42.78 ± 11.89
Shogaol	0.12 ± 0.01	2.71 ± 0.62	6.06 ± 1.11
Gingerol	14.09 ± 3.87	16.73 ± 4.64	76.94 ± 4.13
Crude ethanolic extract of *C. longa*	12.63 ± 1.34	10.07 ± 0.82	38.11 ± 10.75
Crude ethanolic extract of *C. zedoaria*	10.36 ± 1.05	7.62 ± 2.00	28.33 ± 3.32
Curcumin	3.51 ± 0.59	2.08 ± 0.15	3.72 ± 0.58
Demethoxycurcumin	3.69 ± 0.53	5.94 ± 2.02	7.95 ± 0.26
Bisdemethoxycurcumin	7.97 ± 2.07	2.71 ± 0.62	6.90 ± 1.59

Table S10 The antibody used in this study were as follows:

**Antibodies**	**Cat. No.**	**Company**
FITC anti-BrdU Antibody	364104	BioLegend, CA, USA
HRP-conjugated goat anti-rabbit IgG	W401B	Promega, WI, USA
HRP-conjugated goat anti-mouse IgG	W402B	
Rabbit monoclonal anti-CD34 antibody	sc-9095	Santa Cruz Biotechnology, TX, USA
Rabbit polyclonal anti-GAPDH antibody	sc-25778	
Rabbit polyclonal anti-WT1 antibody	sc-192	
Cleaved caspase-3 (Asp175) (5A1E) rabbit mAb	9664	Cell signaling Technology, MA, USA
Cleaved PARP (Asp214) (D64E10) XP® rabbit mAb	5625	
Phospho-Akt (Ser473) (D9E) XP® rabbit mAb	4060	
Phospho-c-Jun (Ser73) (D47G9) XP® rabbit mAb	3270	
Phospho-SAPK/JNK (Thr183/Tyr185) (81E11) rabbit mAb	4668	
YAP/TAZ (D24E4) rabbit mAb	8418	
