# Supplementary material for: Anticancer and cancer preventive activities of shogaol and curcumin from Zingiberaceae family plants in KG-1a leukemic stem cells
Source: BMC Complement Med Ther. 2025 Feb 28;25:87. doi: 10.1186/s12906-025-04829-7 (PMC11869560; doi:10.1186/s12906-025-04829-7)
Supplement: Supplementary file 2 — Supplementary Material 2 [file 12906_2025_4829_MOESM2_ESM.pdf]

Supplementary file: raw data of Western blotting

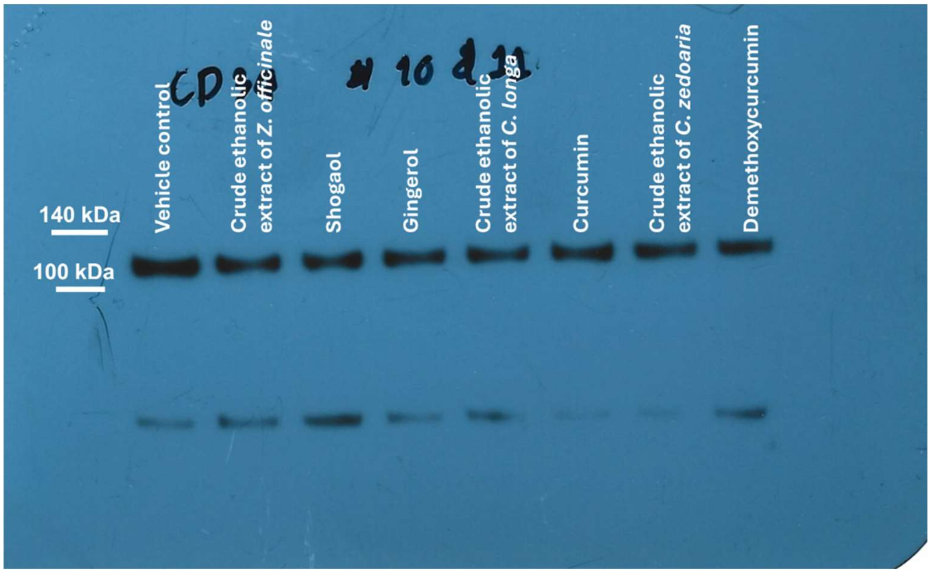

Fig. S14 Raw data showing protein band expression for CD34 after treatment with IC_{20} concentrations of crude ethanolic extracts and their active compounds, compared with the vehicle control in KG-1a cells.

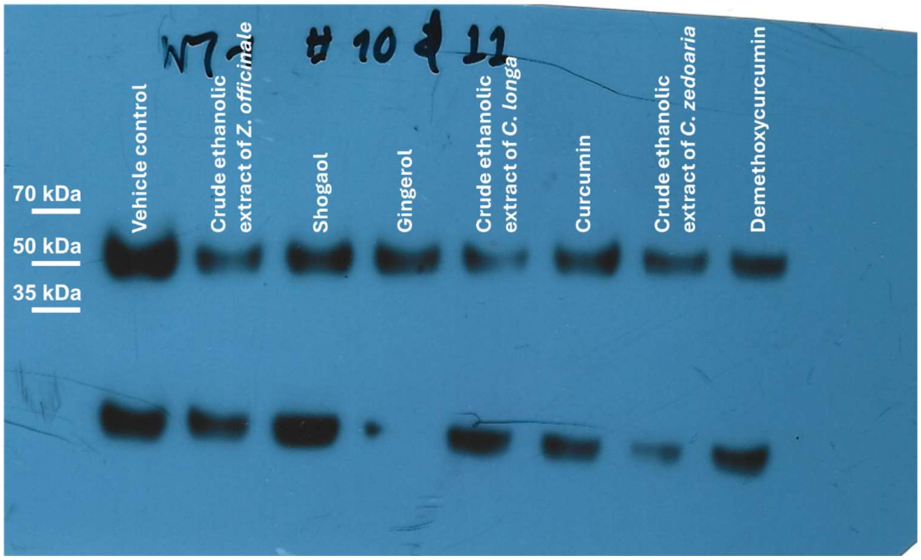

Fig. S15 Raw data showing protein band expression for WT1 after treatment with IC_{20} concentrations of crude ethanolic extracts and their active compounds, compared with the vehicle control in KG-1a cells.

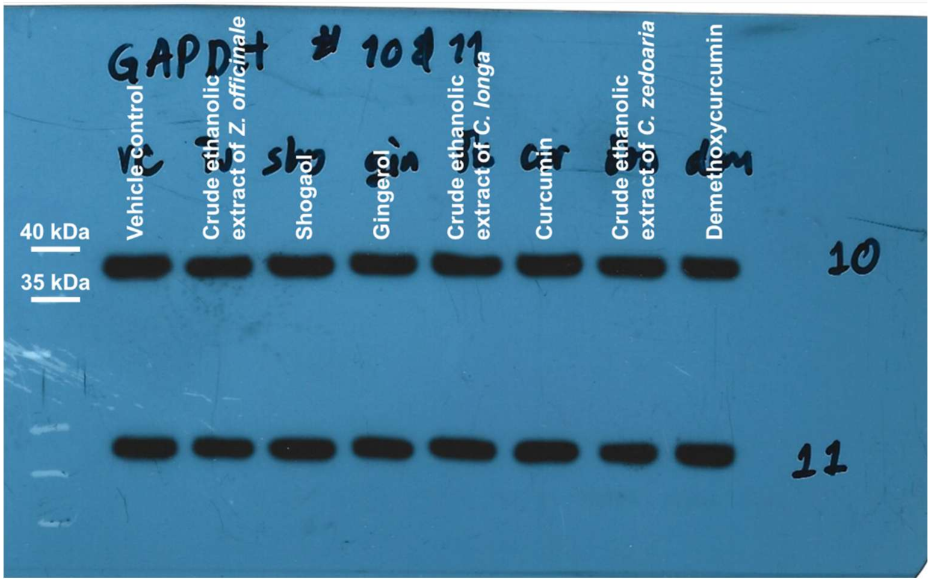

Fig. S16 Raw data showing protein band expression for GAPDH after treatment with IC_{20} concentrations of crude ethanolic extracts and their active compounds, compared with the vehicle control in KG-1a cells.

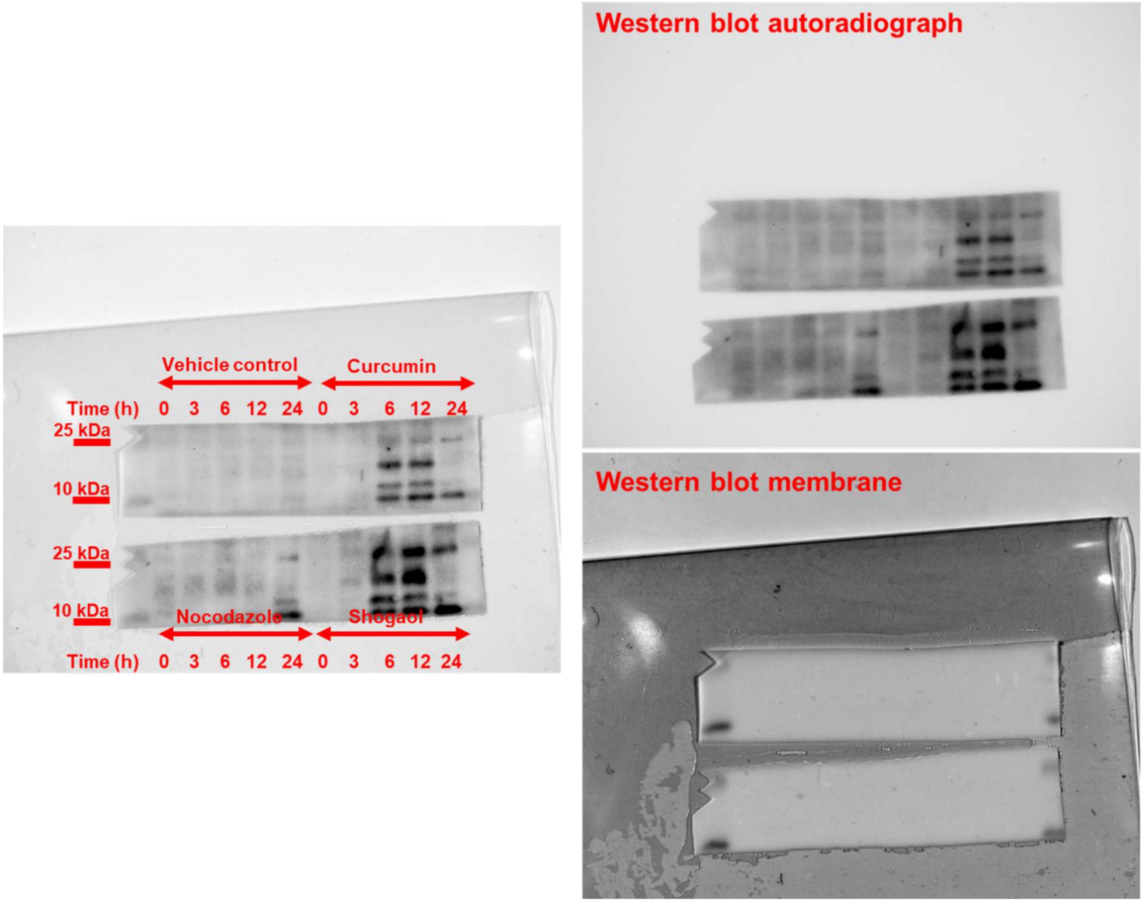

Fig. S17 Raw data showing protein band expression for cleaved caspase-3 in KG-1a cells after incubation with IC_{50} concentrations of curcumin, shogaol, and nocodazole (positive control) for 0–24 h, compared with the vehicle control.

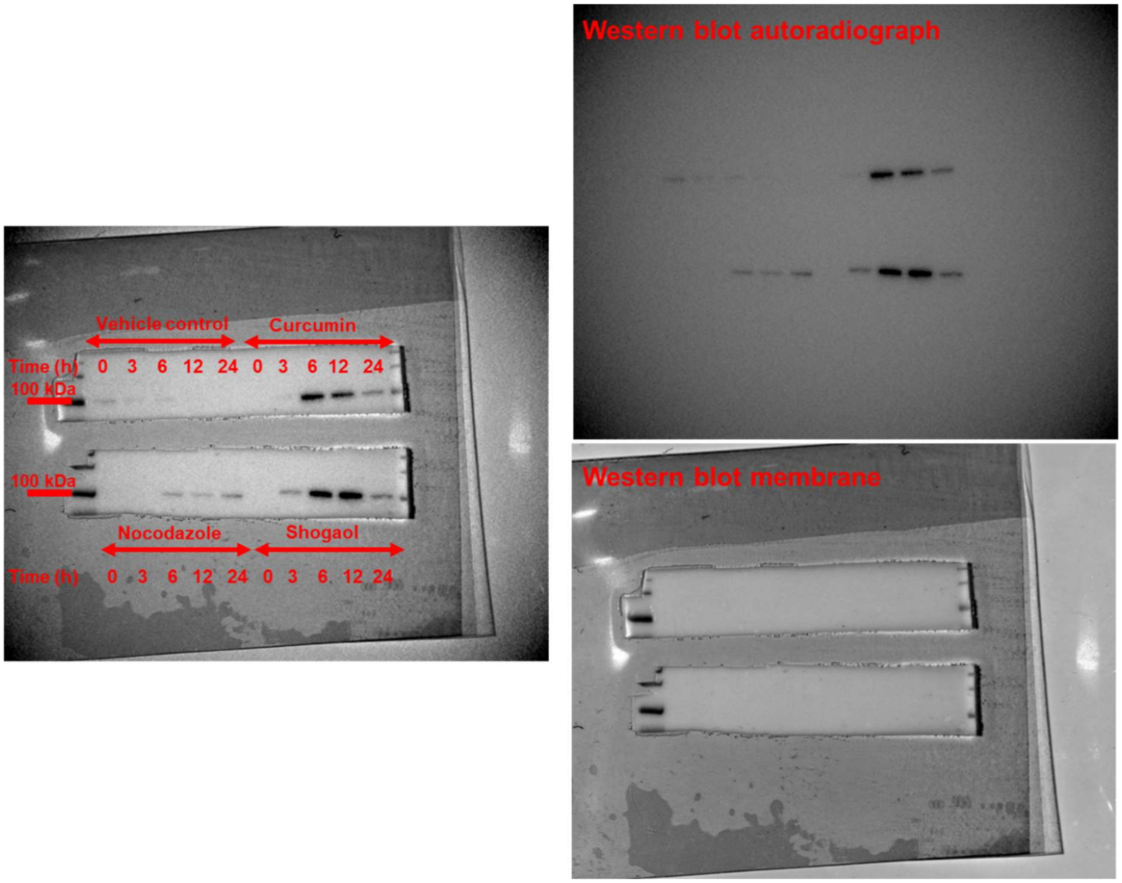

Fig. S18 Raw data showing protein band expression for cleaved PARP in KG-1a cells after incubation with IC_{50} concentrations of curcumin, shogaol, and nocodazole (positive control) for 0–24 h, compared with the vehicle control.

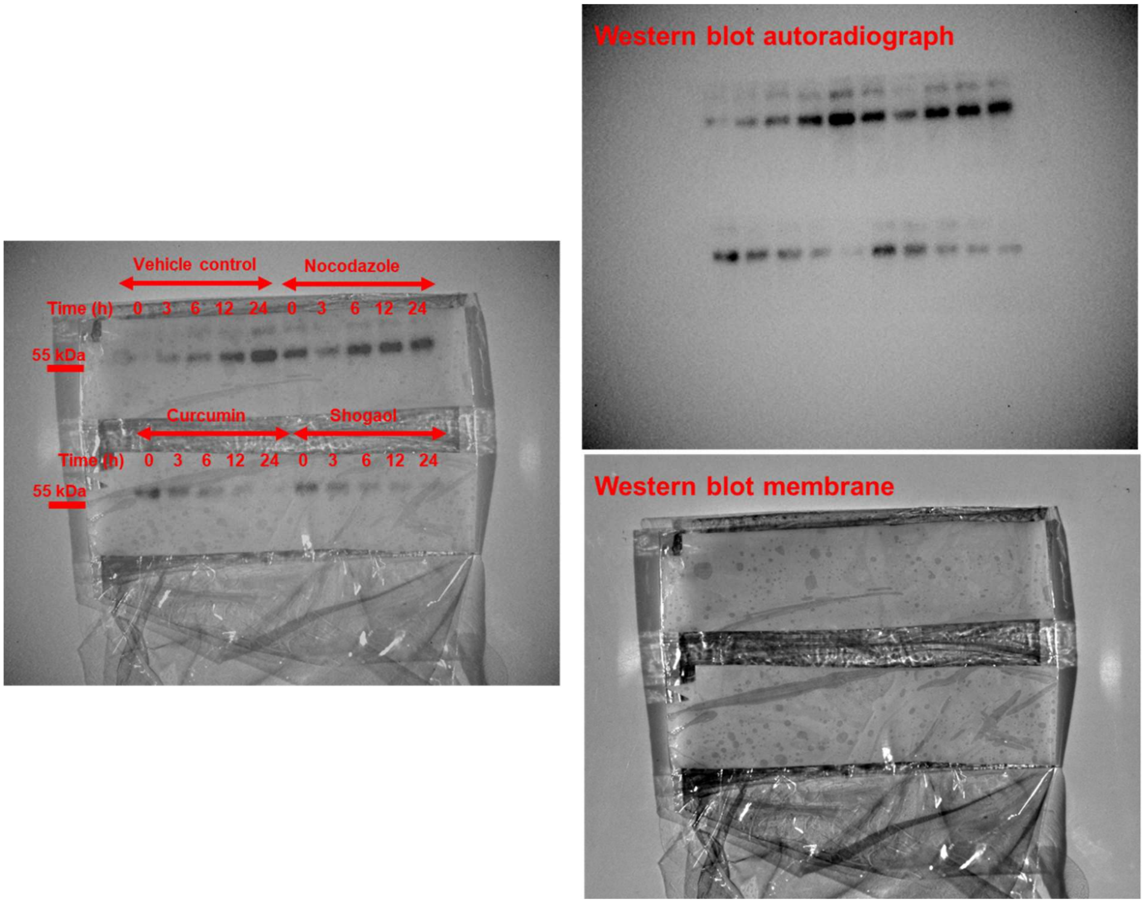

Fig. S19 Raw data showing protein band expression for WT1 in KG-1a cells after incubation with IC_{50} concentrations of curcumin, shogaol, and nocodazole (positive control) for 0–24 h, compared with the vehicle control.

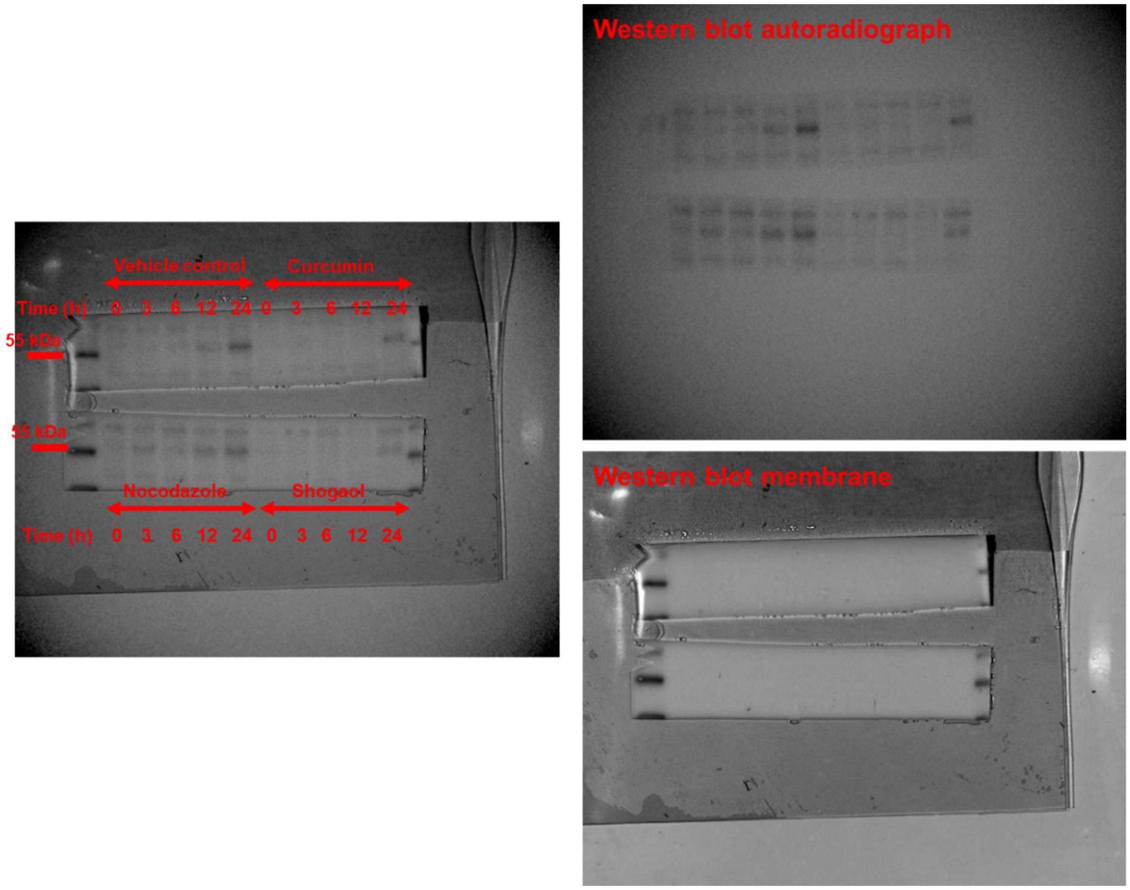

Fig. S20 Raw data showing protein band expression for TAZ in KG-1a cells after incubation with IC_{50} concentrations of curcumin, shogaol, and nocodazole (positive control) for 0–24 h, compared with the vehicle control.

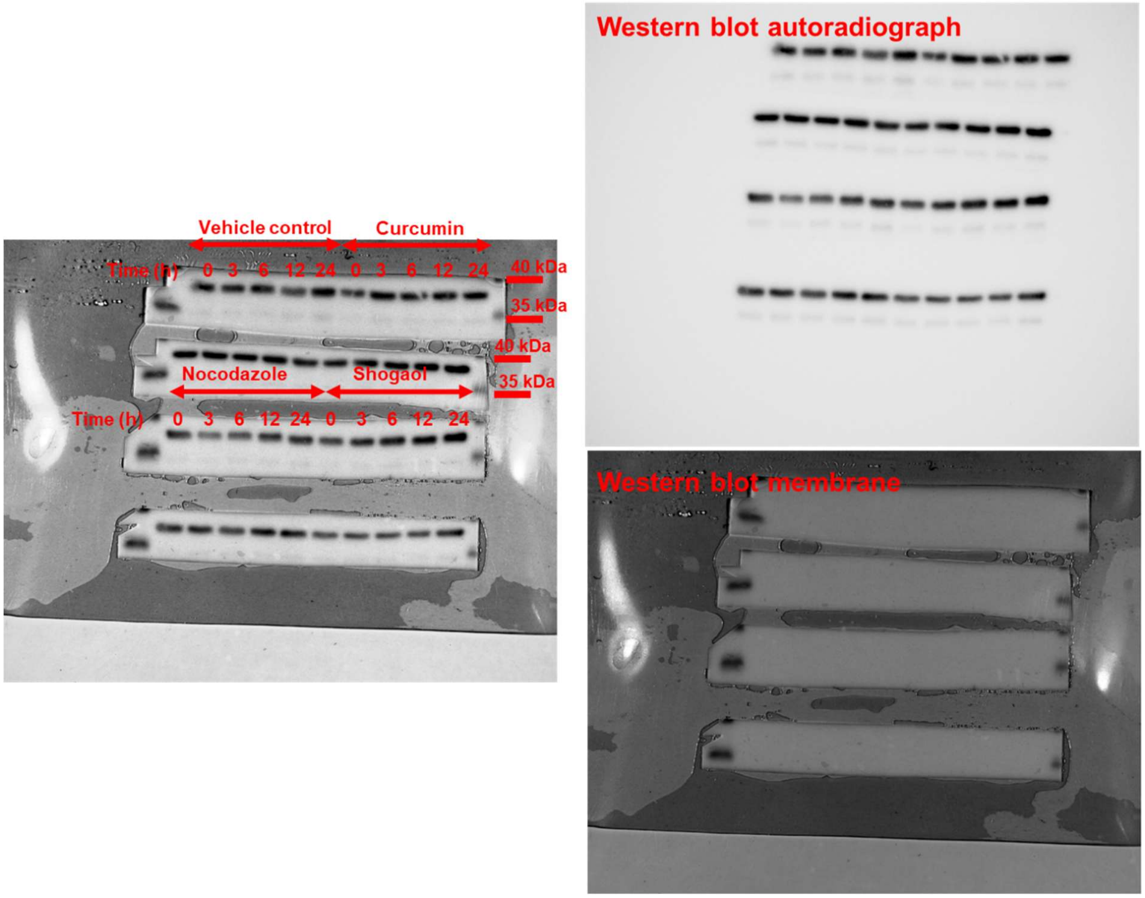

Fig. S21 Raw data showing protein band expression for GAPDH in KG-1a cells after incubation with IC_{50} concentrations of curcumin, shogaol, and nocodazole (positive control) for 0–24 h, compared with the vehicle control.

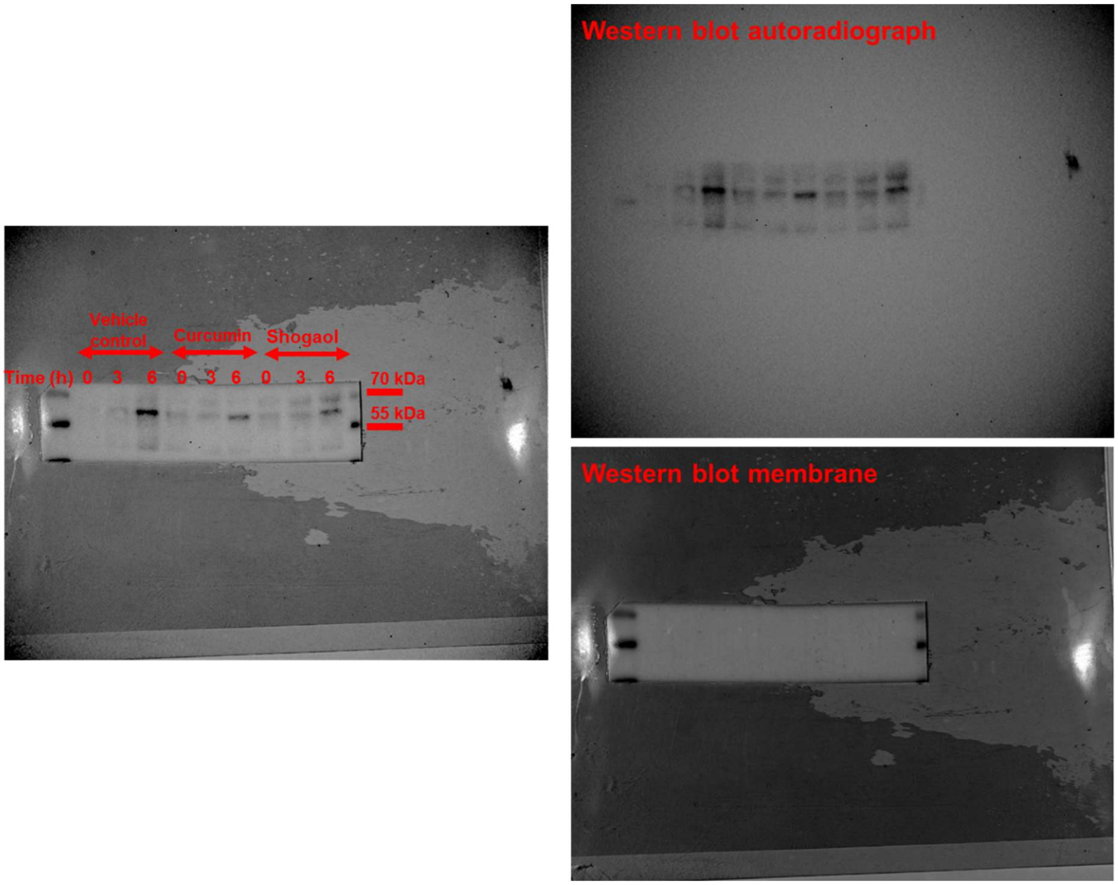

Fig. S22 Raw data showing protein band expression for p-Akt in KG-1a cells after incubation with IC_{50} concentrations of curcumin and shogaol for 0–6 h, compared with the vehicle control.

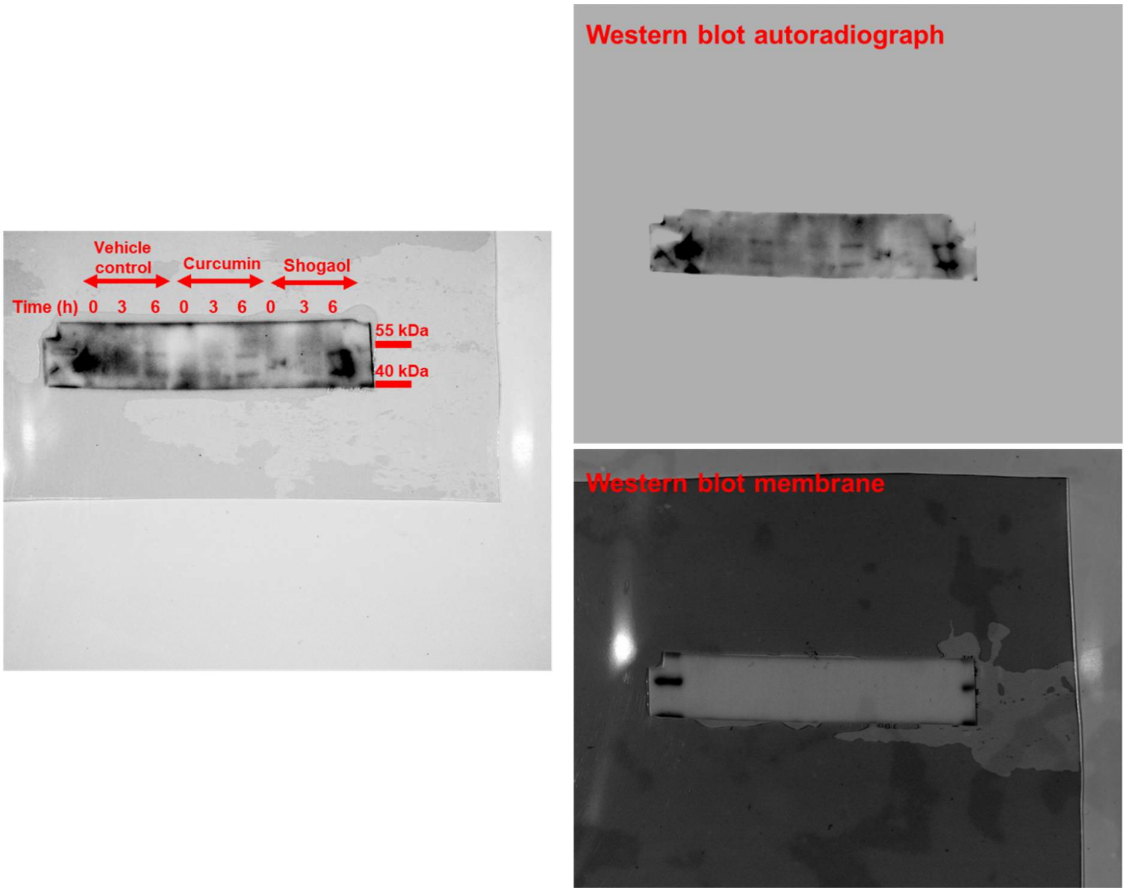

Fig. S23 Raw data showing protein band expression for p-SAPK/JNK in KG-1a cells after incubation with IC_{50} concentrations of curcumin and shogaol for 0–6 h, compared with the vehicle control.

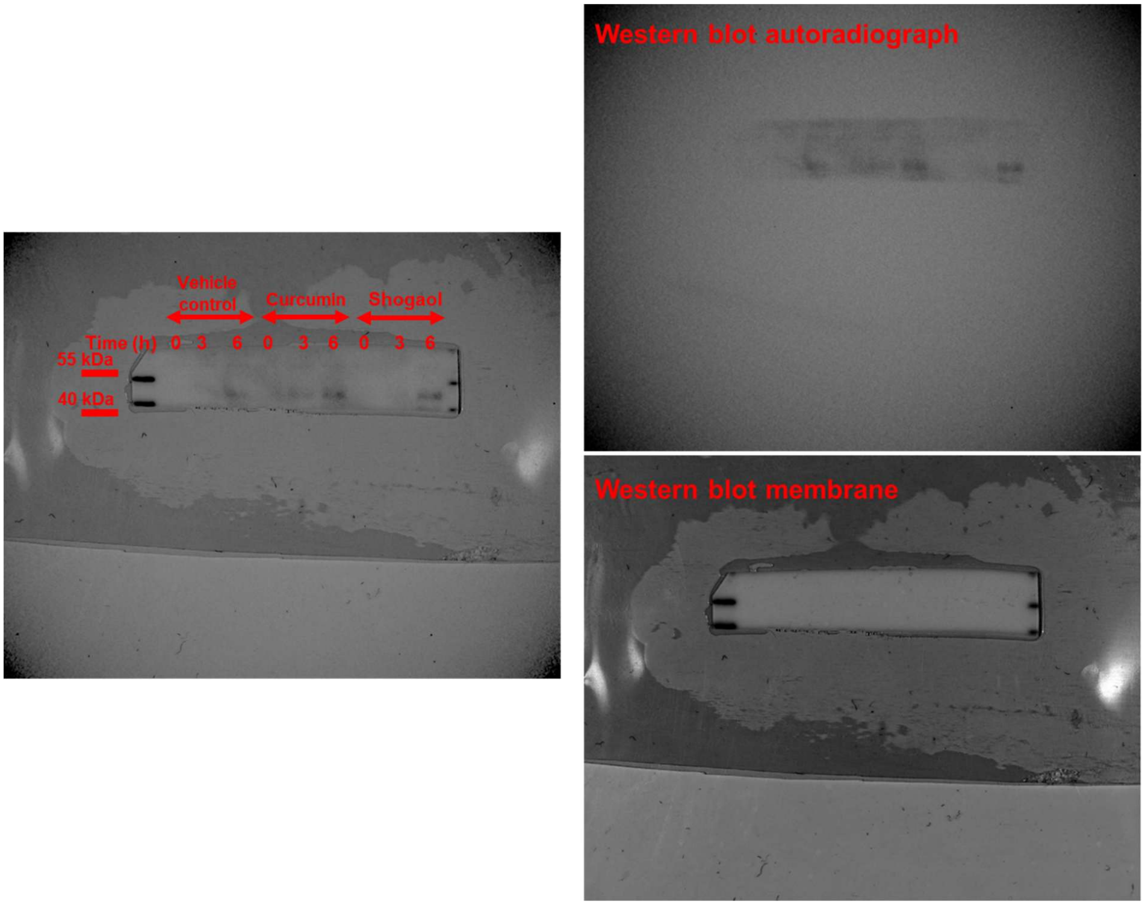

Fig. S24 Raw data showing protein band expression for p-c-Jun in KG-1a cells after incubation with IC_{50} concentrations of curcumin and shogaol for 0–6 h, compared with the vehicle control.

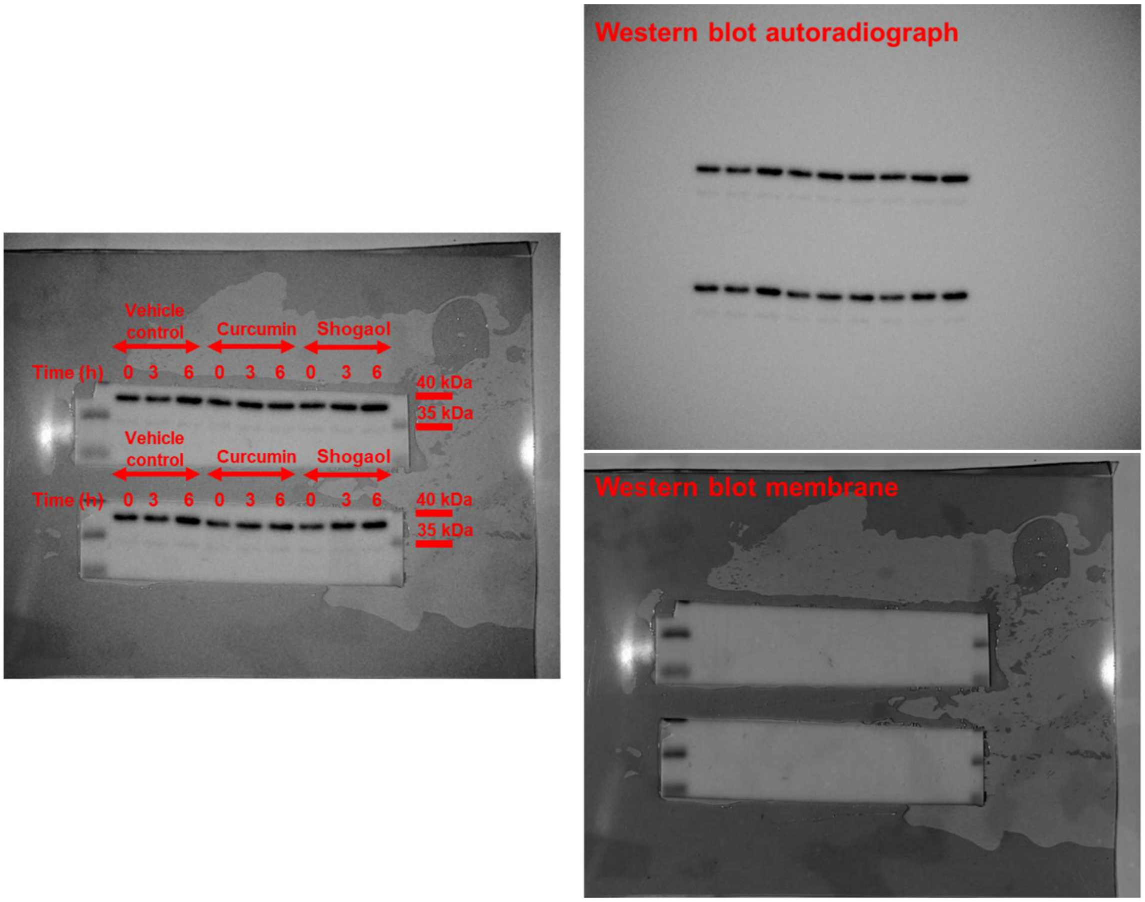

Fig. S25 Raw data showing protein band expression for GAPDH in KG-1a cells after incubation with IC_{50} concentrations of curcumin and shogaol for 0–6 h, compared with the vehicle control.

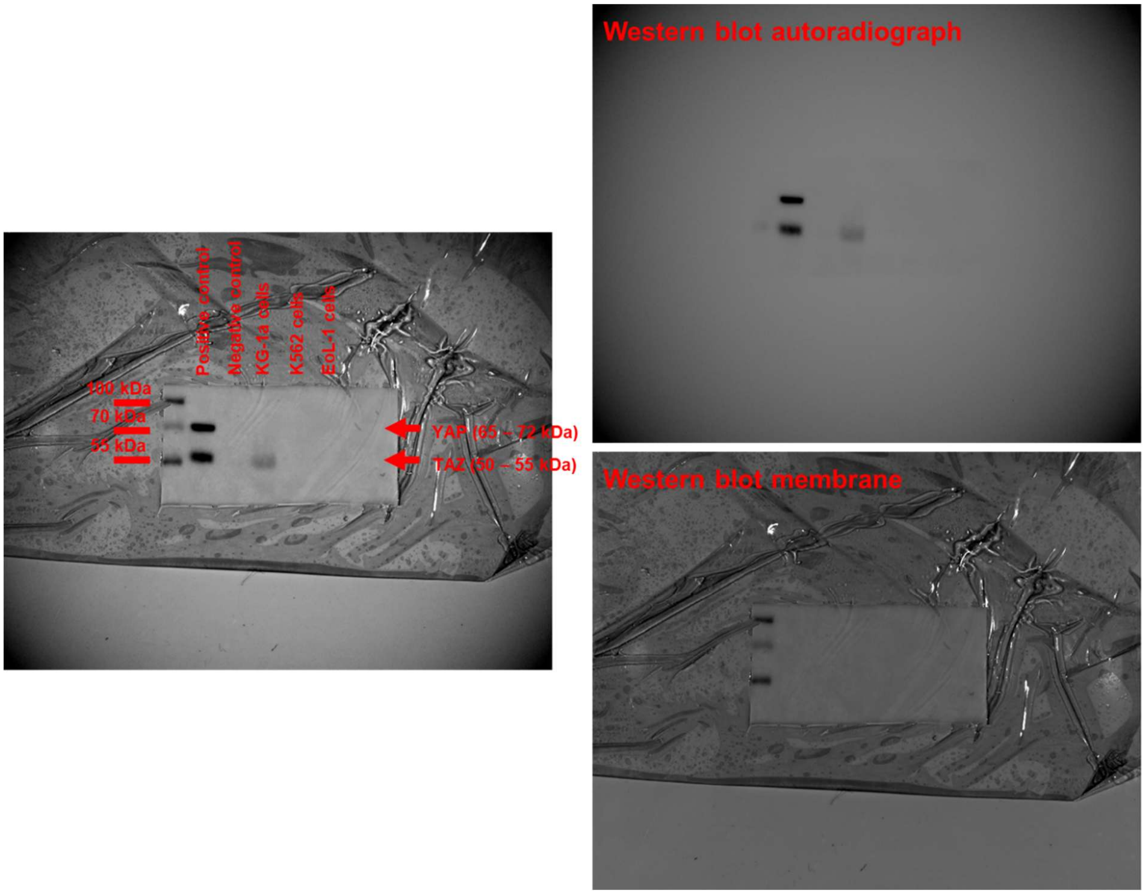

Fig. S26 Raw data showing protein band expression for YAP/TAZ in three leukemic cell lines compared with control. Positive control = wild-type Lewis lung carcinoma cells and negative control = YAP/TAZ knock-out Lewis lung carcinoma cells.

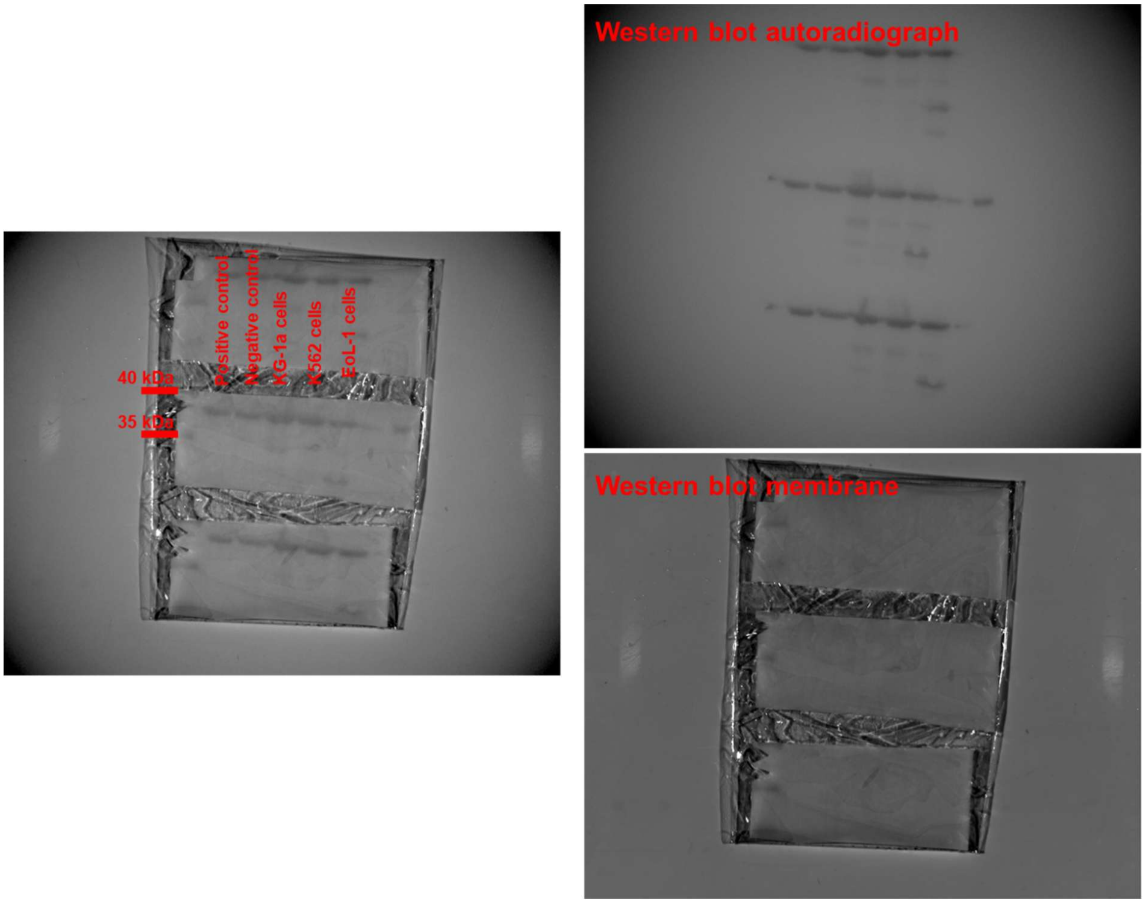

Fig. S27 Raw data showing protein band expression for GAPDH in three leukemic cell lines compared with control. Positive control = wild-type Lewis lung carcinoma cells and negative control = YAP/TAZ knock-out Lewis lung carcinoma cells.
